# Supplementary material for: Adherence to the EAT-Lancet diet and incident depression and anxiety
Source: Nat Commun. 2024 Jul 3;15:5599. doi: 10.1038/s41467-024-49653-8 (PMC11222463; doi:10.1038/s41467-024-49653-8)
Supplement: Supplementary file 1 — Supplementary Information [file 41467_2024_49653_MOESM1_ESM.pdf]

Supplementary materials

Adherence to the EAT-Lancet Diet and Incident Depression and Anxiety

Xujia Lu[1], Luying Wu[1], Liping Shao[1], Yulong Fan[1], Yalong Pei[1], Xinmei Lu[1], Yan Borné[2#], Chaofu Ke[1#*]

[1] Department of Epidemiology and Biostatistics, School of Public Health, Suzhou Medical College of Soochow University, Suzhou, China.

[2] Department of Clinical Sciences Malmö, Lund University, Malmö, Sweden.

[#] These authors jointly supervised this work.

[*] Correspondence should be addressed to: Chaofu Ke, e-mail: cfke@suda.edu.cn.

Supplementary Methods 1 Assessment for the EAT-Lancet and other diet indexes

Supplementary Methods 2 Assessment of covariates

Supplementary Figure 1 The flowchart of participant selection

Supplementary Figure 2 Kaplan-Meier curves for depression (A), anxiety (B) and their co-occurrence (C) stratified by the Knuppel EAT-Lancet diet index; for depression (D), anxiety (E) and their co-occurrence (F) stratified by the Stubbendorff EAT-Lancet diet index; and for depression (G), anxiety (H) and their co-occurrence (I) stratified by the Kesse-Guyot EAT-Lancet diet index

Supplementary Table 1 Baseline characteristics of the study participants according to categories of the Stubbendorff EAT-Lancet index (range, 0-38 points)

Supplementary Table 2 Baseline characteristics of the study participants according to quintiles of the Kesse-Guyot EAT-Lancet index

Supplementary Table 3 Associations between the Knuppel EAT-Lancet diet index and risks of depression and anxiety after excluding participants who only completed the online 24-hour dietary recall questionnaire on 1 occasion (N=110,130)

Supplementary Table 4 Associations between the Stubbendorff EAT-Lancet diet index and risks of depression and anxiety after excluding participants who only completed the online 24-hour dietary recall questionnaire on 1 occasion (N=110,130)

Supplementary Table 5 Associations between the Kesse-Guyot EAT-Lancet diet index and risks of depression and anxiety after excluding participants who only completed the online 24-hour dietary recall questionnaire on 1 occasion (N=110,130)

Supplementary Table 6 Associations between the Knuppel EAT-Lancet diet index and risks of depression and anxiety using follow-up phases which began at the time of the completion of the latest dietary assessment

Supplementary Table 7 Associations between the Stubbendorff EAT-Lancet diet index and risks of depression and anxiety using follow-up phases which began at the time of the completion of the latest dietary assessment

Supplementary Table 8 Associations between the Kesse-Guyot EAT-Lancet diet index and risks of depression and anxiety using follow-up phases which began at the time of the completion of the latest dietary assessment

Supplementary Table 9 Associations between the Knuppel EAT-Lancet diet index and risks of depression and anxiety after excluding depression or anxiety cases that occurred within the first five years of follow-up

Supplementary Table 10 Associations between the Stubbendorff EAT-Lancet diet index and risks of depression and anxiety after excluding depression or anxiety cases that occurred within the first five years of follow-up

Supplementary Table 11 Associations between the Kesse-Guyot EAT-Lancet diet index and risks of depression and anxiety after excluding depression or anxiety cases that occurred within the first five years of follow-up

Supplementary Table 12 Associations of individual food components of the Knuppel EAT-Lancet index with risks of depression and anxiety

Supplementary Table 13 Associations of individual food components of the Stubbendorff EAT-Lancet index with risks of depression and anxiety

Supplementary Table 14 Associations of individual food components of the Kesse-Guyot EAT-Lancet index with risks of depression and anxiety

Supplementary Table 15 Mediating effects of BMI, CVD, T2D and hypertension in the associations between the EAT-Lancet indexes and risks of depression and anxiety

Supplementary Table 16 Subgroup analyses of associations between the Knuppel EAT-Lancet diet index and risks of depression and anxiety

Supplementary Table 17 Subgroup analyses of associations between the Stubbendorff EAT-Lancet diet index and risks of depression and anxiety

Supplementary Table 18 Subgroup analyses of associations between the Kesse-Guyot EAT-Lancet diet index and risks of depression and anxiety

Supplementary Table 19 Baseline characteristics of the analysis population and the total population

Supplementary Table 20 Associations between the EAT-Lancet indexes and risks of depression and anxiety in White

Supplementary Table 21 Definition of portion size and food items used in this study

Supplementary Table 22 Cut-offs for each component of the Knuppel and the Kesse-Guyot EAT-Lancet diet index

Supplementary Table 23 Cut-offs for each component of the Stubbendorff EAT-Lancet diet index

Supplementary Table 24 Proportion of 180,446 participants adhering to the Knuppel EAT-Lancet diet index recommendations

Supplementary Table 25 Proportion of 180,446 participants adhering to the Stubbendorff EAT-Lancet diet index recommendations

Supplementary Table 26 Scores of each individual components of the Kesse-Guyot EAT-Lancet diet index

Supplementary Methods 1

Assessment for the Knuppel EAT-Lancet diet index

The EAT-Lancet score is consisted of 8 main dietary categories, including whole grains, tubers and starchy vegetables, vegetables, fruits, dairy foods, protein sources, added fats, and added sugars. Participants were assigned with a point for meeting each of the recommendations. Each dietary component contributed 0 or 1 point resulting in a total score ranging from 0 to 14 points. The higher dietary scores indicated a greater adherence to the individual healthy eating patterns[1].

The online 24-h dietary assessment tool did not record the concrete weight of consumed food, but the number of predefined portion size was defined using the UK's standard food composition database[2][3] (e.g., how many bowls of cereals, how many serving of Quorn they ate in the last 24 h). We then calculated the quantity by multiplying the portion size by the number of portions consumed for each food item[4].

Assessment for the Stubbendorff EAT-Lancet diet index

An EAT-Lancet diet index was calculated to estimate the adherence to the EAT-Lancet Commission recommendations on healthy diets from sustainable food systems[5][6]. Details of the EAT-Lancet diet index were described in the development article[7]. The EAT-Lancet index is consisted of 14 dietary components, with 7 emphasized components (whole grains, vegetables, fruits, fish, legumes, nuts, and unsaturated oils) and 7 limited components (potatoes, dairy, beef and lamb, pork, poultry, eggs, and added sugar). Participants were assigned with 3 points for the highest adherence and 0 point for the lowest for emphasized components. The scoring pattern was inverted for the limited components. The 24-h dietary recall questionnaire was not able to separate red meat into beef/lamb and pork hence these were aggregated into total red meat intake. As there was limited information about the types of oils in the 24-h dietary recall, participants met the recommendation of ratio of 0.8 for unsaturated: saturated fat intake were assigned with 2 points and those did not meet the recommendation were assigned with 0 point[1]. The boundaries of the

different scores (0, 1, 2, or 3) were based on the target intake levels and the reference intervals[7]. Each dietary component contributing 0-3 points (except for unsaturated oils with 0 or 2 points) constructed the EAT-Lancet index ranging from 0 (worst) to 38 (best) points, with higher scores indicating greater adherence to the recommended eating pattern.

Assessment for the Kesse-Guyot EAT-Lancet diet index

Based on the definition of the universal healthy diet[5], component and cut-off of the EAT-Lancet diet have been already proposed in a previous work[1], also regarding 14 food groups. Cut-offs for each component are presented in Supplementary Table 22. To improve the power of discrimination of the dietary index reflecting the adherence to the EAT-Lancet diet, compared to the previously developed score[1], we accounted for deviation from the cut-off value. The EAT-Lancet diet index for an individual j with intake for each i was computed as follows [8]:

$$\text{Kesse} - \text{Guyot EAT} - \text{Lancet diet index}_i = \frac{100 \times \left\{ \sum_{component\ i=1}^{14} \frac{a_i \times \left(cut\ off_i - \frac{consumption_{ij} \times 2500}{Energy\ intake_j} \right)}{cut\ off_i} \right\}}{14}$$

Where i referred to the 14 food groups and j is the individual. $a_i = 1$ for component to limit and $a_i = -1$ for component to promote[8].

Assessment for other diet indexes

We calculated the plant-based diet index[9], the DASH diet index[10] and the Mediterranean diet index[11][12] as described in previous literatures.

Plant-based diet index

We classified foods and beverages reported in the Oxford WebQ into 17 food groups (whole grains, fruits, vegetables, nuts, legumes and vegetarian protein alternatives, tea and coffee, fruit juices, refined grains, potatoes, sugar-sweetened beverages, sweets and desserts, animal fat, dairy, egg, fish or seafood, meat and miscellaneous animal-based foods). Then, these groups were classified into three classes: healthy

plant foods, less healthy plant foods and animal foods. Food groups (measured in servings per day) were categorized into quintiles, and each was given positive scores (quintile1=1 to quintile5=5) or reverse scores (quintile5=1 to quintile1=5). For calculating the PDI, foods in both plant food groups were assigned positive scores and foods in the animal food group were given reverse scores. For calculating the hPDI, foods in the healthy plant food group were given positive scores while the less healthy plant and animal food groups received reverse scores. For calculating the uPDI, foods in the less healthy plant food group were given positive scores, and foods in the healthy plant and animal food groups received reverse scores. The 17 food group scores for an individual were summed to obtain PDIs and the total score ranged from 17 to 85 points, respectively[9].

DASH diet index

The scoring system of DASH diet score is based on quintiles with the lowest quintile intake receiving one point and the top quintile receiving 5 points for healthy components (vegetables, fruits, nuts, legumes, whole grains, and low-fat dairy products); The scoring for unhealthy components (sugar-sweetened drinks, red and processed meats and estimated 24-hour sodium excretion) is reversely coded so that quintile 1 receives 5 points and quintile 5 receives one point. The total score ranged from 8 to 40 points. Higher scores are related to greater adherence to the DASH diet pattern[10].

Mediterranean diet index

The Mediterranean diet score is a 14-point score developed as part of the Prevención con Dieta Mediterránea (PREDIMED) trial[11]. The Mediterranean diet score is calculated with a binary evaluation for each of the 14 food components, with one point awarded if the participant's consumption meets a pre-defined cut-off, and zero point if they do not. The total possible score ranges from 0–14 points. The Mediterranean diet score awards points for use of olive oil as the main culinary fat and, separately, for consumption of a target amount (4 or more tablespoons per day) of

olive oil. Although we were able to determine use of olive oil as a culinary fat and to award points for consumption (1 point) or non-consumption (0 points) accordingly, it was not possible to determine the amount of olive oil consumed from the available dietary data, limiting the maximum possible scores to 13 points in this study[12].

Supplementary Methods 2

Assessment of covariates

Covariates (except total energy intake) were collected at baseline (2006-2010). Covariates were made up of sociodemographic characteristics, lifestyle factors and other potential confounding factors. Sociodemographic covariates included age (continuous), sex (males and females), ethnicity (classified as White, mixed, Asian/Asian British, Black/Black British, and others), and the Townsend deprivation index. The Townsend deprivation index, derived from the postcode of residence, was used to describe the area-based socioeconomic status through the quartiles of indices[13]. Lifestyle covariates including smoking status (classified as never, former and current smoking) and frequency of alcohol intake (divided into never, special occasions only, one to three times a month, once or twice a week, three or four times a week, and daily) were measured by self-report. Physical activity, the volume of which was calculated as the sum of walking, moderate and vigorous activity over the previous week, measured as metabolic equivalents task (METs min/week)[14], was assessed by the International Physical Activity Questionnaire (IPAQ) short form[15]. Physical activity was categorized into four groups: none, low (MET <600 min/week), moderate (600-3000 MET) and high (≥ 3000 MET). Hypertension was determined by the combination of antihypertensive medication use, records of diagnosis by doctors and clinical measurement of blood pressure (systolic blood pressure (SBP)≥ 140 mmHg or diastolic blood pressure (DBP)≥ 90 mmHg). Height and body weight were measured by well-trained nurses during the initial assessment center visit. Body mass index (BMI, kg/m^2) was calculated as weight (kg) divided by height squared (m^2) and divided into four categories according to the recommendations of the World Health Organization (WHO): underweight (<18.5 kg/m^2), normal weight (18.5 to 25 kg/m^2), overweight (25 to 30 kg/m^2), and obesity (≥ 30 kg/m^2). Total energy intake was assessed by the 24-hour dietary recall questionnaire. If participants completed dietary assessments multiple times, an average total energy intake was calculated. The details of these assessments can be found on the UK Biobank website (www.ukbiobank.ac.uk).

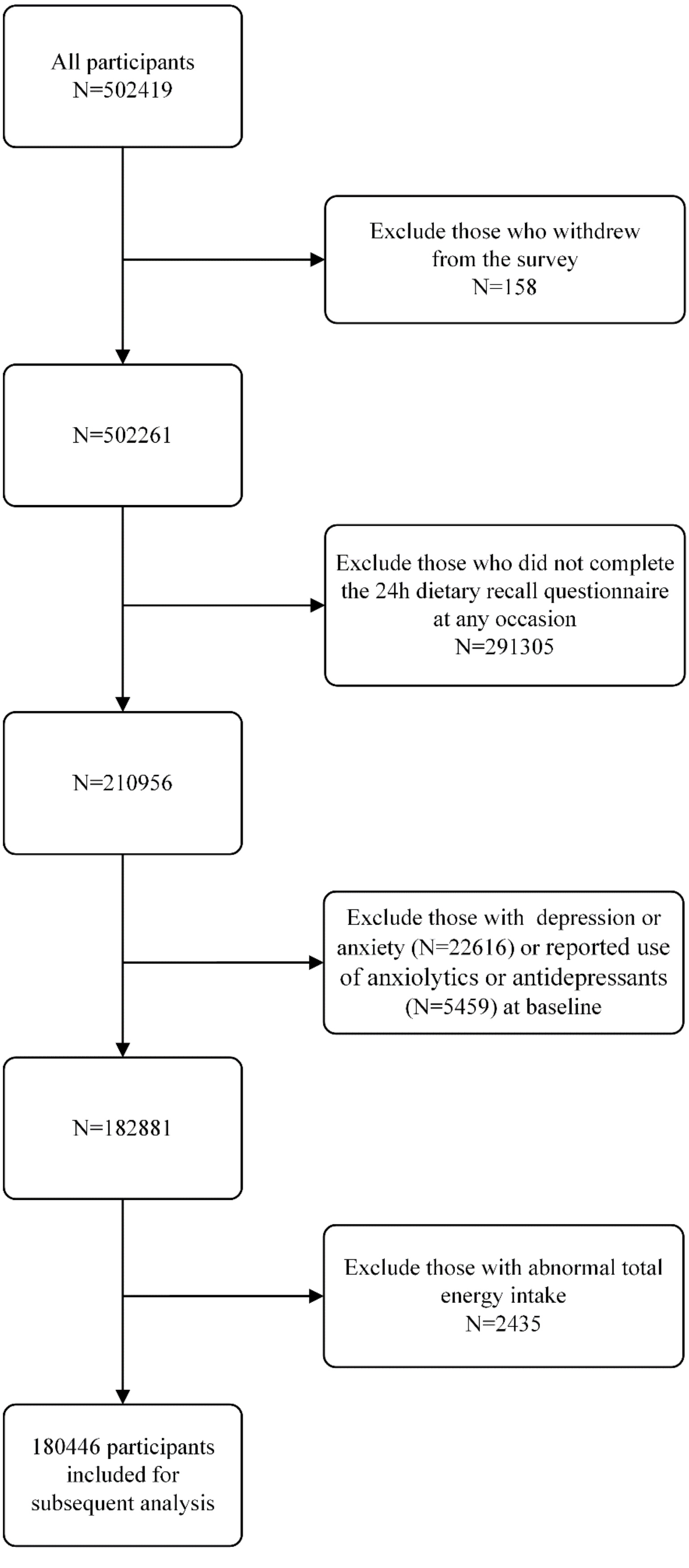

Supplementary Figure 1 The flowchart of participant selection

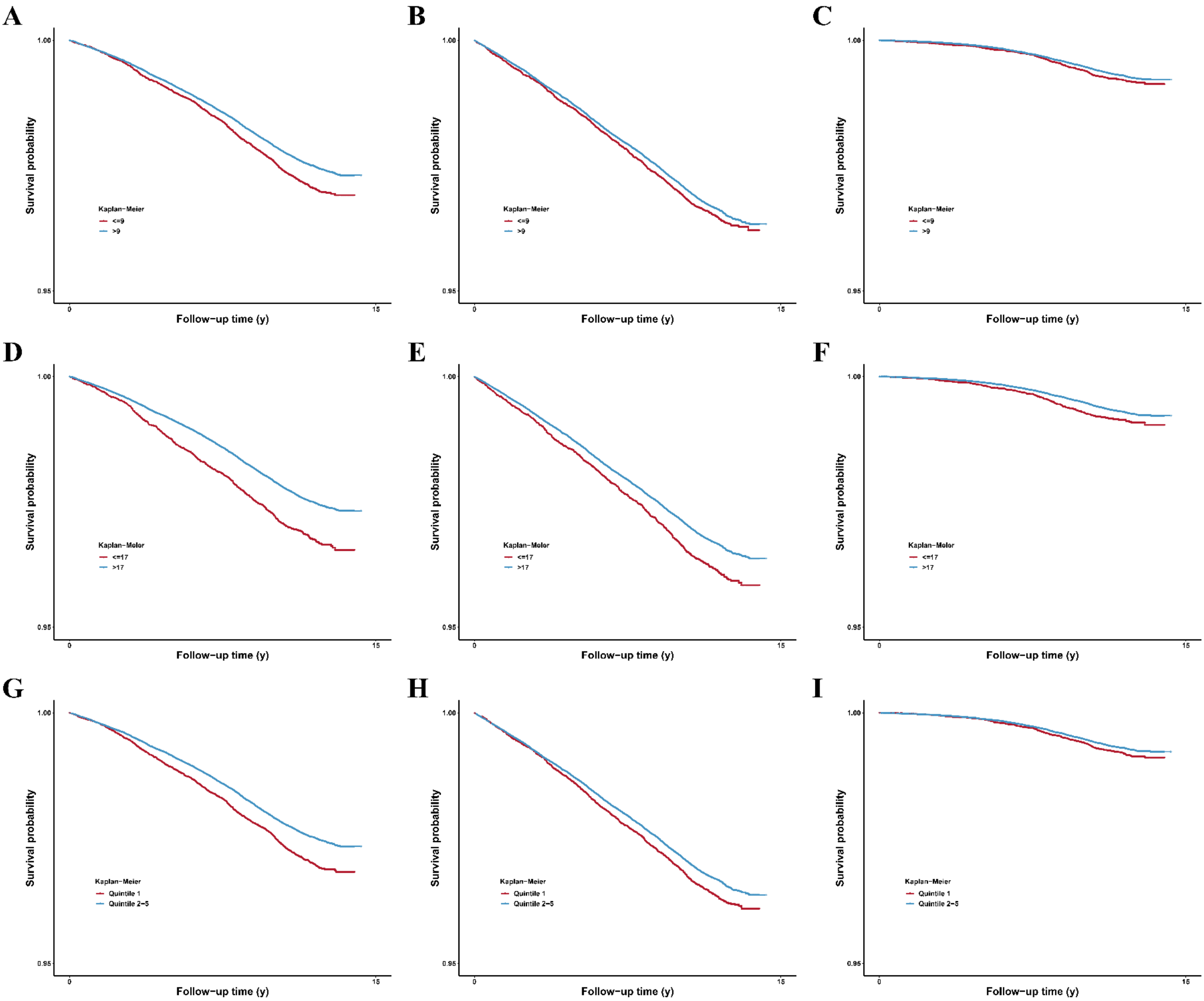

Supplementary Figure 2 Kaplan-Meier curves for depression (A), anxiety (B) and their co-occurrence (C) stratified by the Knuppel EAT-Lancet diet index; for depression (D), anxiety (E) and their co-occurrence (F) stratified by the Stubbendorff EAT-Lancet diet index; and for depression (G), anxiety (H) and their co-occurrence (I) stratified by the Kesse-Guyot EAT-Lancet diet index

Source data are provided as a Source Data file.

Supplementary Table 1 Baseline characteristics of the study participants according to categories of the Stubbendorff EAT-Lancet index (range, 0-38 points)

Characteristics	Total	Categories of the Stubbendorff EAT-Lancet index				
		≤17	18-20	21-23	24-26	≥27
N	180446	20312	40578	55648	43019	20889
Age (years)	56.16±7.97	54.5±8.2	55.7±8.1	56.3±7.9	56.7±7.8	57.0±7.6
Sex (male, %)	83824 (46.45)	12721 (62.63)	21324 (52.55)	25645 (46.08)	16933 (39.36)	7201 (34.47)
Ethnic (%)						
White	172018 (95.33)	19123 (94.15)	38590 (95.10)	53184 (95.57)	41110 (95.56)	20011 (95.80)
Mixed	1057 (0.59)	154 (0.76)	266 (0.66)	300 (0.54)	228 (0.53)	109 (0.52)
Asian or British	2573 (1.43)	259 (1.28)	553 (1.36)	800 (1.44)	688 (1.60)	273 (1.31)
Black or Black British	2278 (1.26)	467 (2.30)	603 (1.49)	609 (1.09)	688 (0.92)	205 (0.98)
Other	1862 (1.03)	227 (1.12)	420 (1.04)	538 (0.97)	453 (1.05)	224 (1.07)
Townsend score (%)						
Above median	90124 (49.95)	11144 (54.86)	20537 (50.61)	27110 (48.72)	20984 (48.78)	10349 (49.54)
Others	90322 (50.05)	9168 (45.14)	20041 (49.39)	28538 (51.28)	22035 (51.22)	10540 (50.46)
Smoking status (%)						
Never smoked	103269 (57.23)	9985 (49.16)	22523 (55.51)	32233 (57.92)	25725 (59.80)	12803 (61.29)
Former smoker	63446 (35.16)	7374 (36.30)	14213 (35.03)	19707 (35.41)	15000 (34.87)	7152 (34.24)
Current smoker	13267 (7.35)	2882 (14.19)	3720 (9.17)	3572 (6.42)	2210 (5.14)	883 (4.23)
Alcohol intake (%)						
Never	10376 (5.75)	1104 (5.44)	2265 (5.58)	3189 (5.73)	2502 (5.82)	1316 (6.30)
Special occasions only	16955 (9.40)	1837 (9.04)	3767 (9.28)	5088 (9.14)	4088 (9.50)	2175 (10.41)
One to three times a month	19500 (10.81)	2158 (10.62)	4423 (10.90)	5866 (10.54)	4683 (10.89)	2370 (11.35)
Once or twice a week	45303 (25.11)	4856 (23.91)	10223 (25.19)	14184 (25.49)	10835 (25.19)	5205 (24.92)
Three or four times a week	46353 (25.69)	4955 (24.39)	10174 (25.07)	14394(25.87)	11362 (26.41)	5468 (26.18)
Daily or almost daily	41828 (23.18)	5379 (26.48)	9692 (23.88)	12887 (23.16)	9525 (22.14)	4345 (20.80)
Physical activity (%)						
None	2216 (1.23)	416 (2.05)	627 (1.55)	644 (1.16)	394 (0.92)	135 (0.65)
Low	24885 (13.79)	3341 (16.45)	6229 (15.35)	7751 (13.93)	5310 (12.34)	2254 (10.79)
Moderate	82185 (45.55)	8500 (41.85)	18196 (44.84)	25572 (45.95)	20104 (46.73)	9813 (46.98)
High	44204 (24.50)	4821 (23.73)	9306 (22.93)	13307 (23.91)	10947 (25.45)	5823 (27.88)
Hypertension (%)	76077 (42.16)	9025 (44.43)	17448 (43.00)	23679 (42.55)	17614 (40.94)	8311 (39.79)
Body mass index (kg/m^2)	26.82±4.53	27.93±4.77	27.36±4.61	26.86±4.49	26.29±4.34	25.71±4.20
Total energy intake (kcal/day)	2047±543	2219±612	2087±561	2033±532	1991±505	1954±491
Whole grains (g/day)*	86.7 (0.0, 173)	0.0 (0.0, 65.0)	43.3 (0.0, 173)	86.7 (0.0, 173)	86.7 (0.0, 173)	144 (57.8, 173)
Potatoes (g/day)	29.0 (0.0, 58.0)	29.0 (0.0, 58.0)	29.0 (0.0, 58.0)	29.0 (0.0, 58.0)	29.0 (0.0, 50.7)	29.0 (0.0, 43.5)
Vegetables (g/day)	200 (100, 350)	100 (0.0, 200)	150 (62.5, 275)	200 (100, 344)	263 (150, 400)	342 (225, 475)
Fruits (g/day)	245 (133, 381)	48 (0.0, 150)	150 (74, 300)	249 (150, 382)	300 (198, 443)	348 (250, 486)
Dairy (g/day)**	125 (35.0, 200)	93.3 (0.0, 210)	113 (0.0, 199)	126 (40.0, 199)	135 (52.5, 202)	140 (57.5, 195)
Red meat (g/day)	40.0 (0.0, 80.0)	80.0 (40.0, 100)	60.0 (26.7, 80.0)	40.0 (0.0, 80.0)	20.0 (0.0, 50.0)	0.0 (0.0, 26.7)
Poultry (g/day)	0.0 (0.0, 40.0)	0.0 (0.0-60.0)	0.0 (0.0, 40.0)	0.0 (0.0, 40.0)	0.0 (0.0, 26.7)	0.0 (0.0, 20.0)
Eggs (g/day)	0.0 (0.0, 30.0)	30.0 (0.0, 60.0)	0.0 (0.0, 40.0)	0.0 (0.0, 24.0)	0.0 (0.0, 15.0)	0.0 (0.0, 0.0)
Fish (g/day)	0.0 (0.0, 50.0)	0.0 (0.0, 0.0)	0.0 (0.0, 0.0)	0.0 (0.0, 33.3)	33.3 (0.0, 66.7)	50.0 (33.3, 100)
Legumes (g/day)	0.0 (0.0, 25.0)	0.0 (0.0, 0.0)	0.0 (0.0, 12.5)	0.0 (0.0, 25.0)	0.0 (0.0, 33.3)	16.7 (0.0, 50.0)
Nuts (g/day)	0.0 (0.0, 0.0)	0.0 (0.0, 0.0)	0.0 (0.0, 0.0)	0.0 (0.0, 0.0)	0.0 (0.0, 0.0)	0.0 (0.0, 14.0)
Unsaturated fat : saturated fat	1.5 (1.2, 1.9)	1.4 (1.1, 1.8)	1.4 (1.2, 1.8)	1.5 (1.2, 1.8)	1.6 (1.3, 1.9)	1.8 (1.4, 2.2)
Added sugar (g/day)	54.2 (35.8, 77.3)	72.4 (48.4, 101)	61.2 (40.5, 84.1)	53.9 (36.1, 75.6)	49.2 (32.8, 68.9)	43.4 (27.7, 59.8)

Data were presented as frequency (%), mean ± standard deviation or median (interquartile range).

* Definition of whole grains in the Stubbendorff EAT-Lancet diet index excludes refined products.

** Intakes of different dairy products were weighted differently according to [Reference 7].

Supplementary Table 2 Baseline characteristics of the study participants according to quintiles of the Kesse-Guyot EAT-Lancet index

Characteristics	Total	Categories of the Kesse-Guyot EAT-Lancet index				
		Quintile 1	Quintile 2	Quintile 3	Quintile 4	Quintile 5
N	180446	36090	36088	36089	36090	36089
Age (years)	56.16±7.97	55.23±8.21	55.94±8.09	56.36±7.93	56.58±7.85	56.70±7.67
Sex (male , %)	83824 (46.45)	22645 (62.75)	19375 (53.69)	16799 (46.55)	14188 (39.31)	10817 (29.97)
Ethnic (%)						
White	172018 (95.33)	34416 (95.36)	34806 (96.45)	34680 (96.10)	34477 (95.53)	33639 (93.21)
Mixed	1057 (0.59)	239 (0.66)	199 (0.55)	194 (0.54)	204 (0.57)	221 (0.61)
Asian or British	2573 (1.43)	338 (0.94)	335 (0.93)	399 (1.11)	568 (1.57)	933 (2.59)
Black or Black British	2278 (1.26)	630 (1.75)	354 (0.98)	348 (0.96)	354 (0.98)	592 (1.64)
Other	1862 (1.03)	335 (0.93)	268 (0.74)	338 (0.94)	367 (1.02)	554 (1.54)
Townsend score (%)						
Above median	90124 (49.95)	18648 (51.67)	17511 (48.52)	17429 (48.29)	17846 (49.45)	18690 (51.79)
Others	90322 (50.05)	17442(48.33)	18577 (51.48)	18660 (51.71)	18244 (5.055)	17399 (48.21)
Smoking status (%)						
Never smoked	103269 (57.23)	18690 (51.79)	20529 (56.89)	21181 (58.69)	21277 (58.96)	21592 (59.83)
Former smoker	63446 (35.16)	12868 (35.66)	12680 (35.14)	12556 (34.79)	12697 (35.18)	12645 (35.04)
Current smoker	13267 (7.35)	4436 (12.29)	2792 (7.74)	2261 (6.27)	2028 (5.62)	1750 (4.85)
Alcohol intake (%)						
Never	10376 (5.75)	1957 (5.42)	1716 (4.76)	1732 (4.80)	1994 (5.53)	2977 (8.25)
Special occasions only	16955 (9.40)	3245 (8.99)	2911 (8.07)	3047 (8.44)	3417 (9.47)	4335 (12.01)
One to three times a month	19500 (10.81)	3869 (10.72)	3678 (10.19)	3725 (10.32)	3823 (10.59)	4405 (12.21)
Once or twice a week	45303 (25.11)	8895 (24.65)	8768 (24.30)	9086 (25.18)	9178 (25.43)	9376 (25.98)
Three or four times a week	46353 (25.69)	8895 (24.65)	9544 (26.45)	9632 (26.69)	9617 (26.65)	8665 (24.01)
Daily or almost daily	41828 (23.18)	9202 (25.50)	9442 (26.16)	8837 (24.49)	8046 (22.29)	6301 (17.46)
Physical activity (%)						
None	2216 (1.23)	722 (2.00)	454 (1.26)	424 (1.17)	311 (0.86)	305 (0.85)
Low	24885 (13.79)	6008 (16.65)	5578 (15.46)	5034 (13.95)	4450 (12.33)	3815 (10.57)
Moderate	82185 (45.55)	15540 (43.06)	16581 (45.95)	16754 (46.42)	16957 (46.99)	16353 (45.31)
High	44204 (24.50)	8071 (22.36)	9240 (22.89)	8556 (23.71)	9125 (25.28)	10230 (28.35)
Hypertension (%)	76077 (42.16)	16217 (44.93)	15351 (42.54)	15356 (42.55)	14699 (40.73)	14454 (40.05)
Body mass index (kg/m^2)	26.82±4.53	27.74±4.71	27.02±4.17	26.67±4.40	26.41±4.39	26.26±4.52
Total energy intake (kcal/day)	2047±543	2188±582	2168±533	2102±509	1998±491	1777±488
Whole grains (g/day)	130 (65.0, 162)	130 (32.5, 130)	130 (65.0, 162)	130 (65.0, 173)	130 (65.0, 173)	130 (65.0, 173)
Potatoes (g/day)	29.0 (0.0, 58.0)	29.0 (0.0, 58.0)	29.0 (7.3, 58.0)	29.0 (0.0, 58.0)	29.0 (0.0, 58.0)	19.3 (0.0, 43.5)
Vegetables (g/day)	200 (100, 350)	118 (37.5, 218)	175 (93.7, 275)	200 (100, 342)	262 (150, 400)	350 (200, 537)
Fruits (g/day)	245 (133, 381)	75 (0.0, 158)	160 (85, 262)	244 (150, 334)	317 (224, 444)	450 (323, 600)
Dairy (g/day)	49.0 (7.0, 155)	28.0 (0.0, 129)	42.7 (7.0, 138)	56 (10.5, 149)	60.0 (14.0, 170)	69 (7.0, 180)
Red meat (g/day)	40.0 (0.0, 80.0)	80.0 (40.0, 120)	53.3 (20.0, 80.0)	40.0 (0.0, 70.0)	25.0 (0.0, 53.3)	0.0 (0.0, 40.0)
Poultry (g/day)	0.0 (0.0, 40.0)	0.0 (0.0, 40.0)	0.0 (0.0, 40.0)	0.0 (0.0, 40.0)	0.0 (0.0, 40.0)	0.0 (0.0, 40.0)
Eggs (g/day)	0.0 (0.0, 30.0)	20.0 (0.0, 60.0)	0.0 (0.0, 30.0)	0.0 (0.0, 20.0)	0.0 (0.0, 15.0)	0.0 (0.0, 0.0)
Fish (g/day)	0.0 (0.0, 50.0)	0.0 (0.0, 33.3)	0.0 (0.0, 50.0)	0.0 (0.0, 50.0)	0.0 (0.0, 50.0)	0.0 (0.0, 50.0)
Legumes (g/day)	0.0 (0.0, 25.0)	0.0 (0.0, 45.0)	0.0 (0.0, 31.2)	0.0 (0.0, 25.0)	0.0 (0.0, 25.0)	0.0 (0.0, 12.5)
Nuts (g/day)	0.0 (0.0, 0.0)	0.0 (0.0, 0.0)	0.0 (0.0, 0.0)	0.0 (0.0, 0.0)	0.0 (0.0, 3.5)	0.0 (0.0, 9.3)
Unsaturated fat : saturated fat	1.5 (1.2, 1.9)	1.4 (1.1, 1.6)	1.4 (1.2, 1.7)	1.5 (1.2, 1.8)	1.6 (1.3, 2.0)	1.8 (1.4, 2.3)
Added sugar (g/day)	54.2 (35.8, 77.3)	73.4 (49.2, 102)	64.5 (45.3, 86.5)	56.8 (40.1, 76.2)	48.7 (34.0, 66.4)	35.9 (22.6, 51.7)

Data were presented as frequency (%), mean ± standard deviation or median (interquartile range).

Supplementary Table 3 Associations between the Knuppel EAT-Lancet diet index and risks of depression and anxiety after excluding participants who only completed the online 24-hour dietary recall questionnaire on 1 occasion (N=110,130)

	N_{case}/N_{total}	Model 0[1]		Model 1[2]		Model 2[3]	
		HR (95% CI)	*P* value	HR (95% CI)	*P* value	HR (95% CI)	*P* value
Depression							
EAT-Lancet diet index categories							
≤9	503/19743	REF		REF		REF	
=10	735/33725	0.853 (0.762-0.955)	0.006	0.831 (0.742-0.932)	0.002	0.884 (0.788-0.991)	0.035
=11	799/36506	0.856 (0.765-0.956)	0.006	0.803 (0.716-0.899)	<0.001	0.884 (0.788-0.992)	0.035
≥12	415/20156	0.803 (0.705-0.915)	0.001	0.713 (0.624-0.814)	<0.001	0.809 (0.706-0.927)	0.002
P for trend	-	0.002		<0.001		0.005	
1-point increment in diet score	2452/110130	0.950 (0.918-0.984)	0.004	0.918 (0.886-0.952)	<0.001	0.954 (0.920-0.989)	0.011
Anxiety							
EAT-Lancet diet index categories							
≤9	620/19743	REF		REF		REF	
=10	1052/33725	0.992 (0.898-1.095)	0.866	0.936 (0.848-1.035)	0.197	0.965 (0.873-1.067)	0.488
=11	1105/36506	0.961 (0.871-1.060)	0.425	0.856 (0.775-0.946)	0.002	0.892 (0.806-0.987)	0.026
≥12	611/20156	0.962 (0.860-1.076)	0.498	0.804 (0.717-0.901)	<0.001	0.839 (0.747-0.943)	0.003
P for trend	-	0.360		<0.001		<0.001	
1-point increment in diet score	3388/110130	0.996 (0.966-1.026)	0.774	0.945 (0.916-0.974)	<0.001	0.957 (0.927-0.987)	0.006
Co-occurrence							
EAT-Lancet diet index categories							
≤9	129/19743	REF		REF		REF	
=10	214/33725	0.969 (0.779-1.206)	0.777	0.932 (0.748-1.161)	0.528	0.984 (0.789-1.227)	0.888
=11	211/36506	0.882 (0.708-1.097)	0.260	0.807 (0.646-1.009)	0.060	0.877 (0.700-1.098)	0.253
≥12	105/20156	0.793 (0.613-1.026)	0.078	0.681 (0.524-0.886)	0.004	0.747 (0.571-0.977)	0.033
P for trend	-	0.046		0.001		0.018	
1-point increment in diet score	659/110130	0.949 (0.887-1.015)	0.126	0.908 (0.848-0.973)	0.007	0.935 (0.871-1.003)	0.060

[1]Model 0: unadjusted.

[2]Model 1: adjusted for age, sex, Townsend scores and ethnicity.

[3]Model 2: adjusted for model 1 plus smoking status, alcohol intake, physical activity, hypertension, BMI and total energy intake.

Statistical tests were two-sided and *P* value of <0.05 was considered statistically significant. No adjustments were made for multiple comparisons.

Supplementary Table 4 Associations between the Stubbendorff EAT-Lancet diet index and risks of depression and anxiety after excluding participants who only completed the online 24-hour dietary recall questionnaire on 1 occasion (N=110,130)

	N_{case}/N_{total}	Model 0[1]		Model 1[2]		Model 2[3]	
		HR (95% CI)	*P* value	HR (95% CI)	*P* value	HR (95% CI)	*P* value
Depression							
EAT-Lancet diet index categories							
≤17	253/9043	REF		REF		REF	
18-20	559/22469	0.888 (0.766-1.031)	0.119	0.866 (0.746-1.005)	0.058	0.933 (0.803-1.083)	0.360
21-23	763/34891	0.779 (0.675-0.898)	<0.001	0.743 (0.643-0.858)	<0.001	0.839 (0.725-0.970)	0.017
24-26	614/29646	0.736 (0.636-0.853)	<0.001	0.685 (0.590-0.795)	<0.001	0.803 (0.691-0.935)	0.005
≥27	263/14081	0.662 (0.557-0.787)	<0.001	0.601 (0.504-0.716)	<0.001	0.727 (0.608-0.869)	<0.001
P for trend	-	<0.001		<0.001		<0.001	
1-point increment in diet score	2452/110130	0.970 (0.960-0.981)	<0.001	0.963 (0.952-0.974)	<0.001	0.978 (0.966-0.989)	<0.001
Anxiety							
EAT-Lancet diet index categories							
≤17	295/9043	REF		REF		REF	
18-20	710/22469	0.970 (0.846-1.111)	0.656	0.915 (0.798-1.048)	0.200	0.952 (0.830-1.091)	0.478
21-23	1085/34891	0.953 (0.838-1.084)	0.465	0.864 (0.758-0.984)	0.027	0.917 (0.805-1.046)	0.197
24-26	877/29646	0.905 (0.793-1.033)	0.138	0.790 (0.691-0.903)	<0.001	0.852 (0.744-0.976)	0.021
≥27	421/14081	0.914 (0.788-1.061)	0.237	0.774 (0.665-0.900)	<0.001	0.840 (0.720-0.979)	0.026
P for trend	-	0.078		<0.001		0.002	
1-point increment in diet score	3388/110130	0.993 (0.984-1.003)	0.169	0.980 (0.971-0.990)	<0.001	0.986 (0.977-0.996)	0.007
Co-occurrence							
EAT-Lancet diet index categories							
≤17	66/9043	REF		REF		REF	
18-20	146/22469	0.892 (0.667-1.192)	0.439	0.857 (0.640-1.148)	0.301	0.918 (0.685-1.231)	0.568
21-23	212/34891	0.832 (0.631-1.096)	0.191	0.777 (0.588-1.028)	0.077	0.869 (0.655-1.152)	0.329
24-26	168/29646	0.775 (0.583-1.030)	0.079	0.702 (0.526-0.938)	0.017	0.812 (0.605-1.089)	0.165
≥27	67/14081	0.649 (0.462-0.912)	0.013	0.572 (0.405-0.807)	0.002	0.673 (0.474-0.956)	0.027
P for trend	-	0.006		<0.001		0.016	
1-point increment in diet score	659/110130	0.972 (0.951-0.993)	0.009	0.962 (0.941-0.983)	<0.001	0.975 (0.953-0.997)	0.025

[1]Model 0: unadjusted.

[2]Model 1: adjusted for age, sex, Townsend scores and ethnicity.

[3]Model 2: adjusted for model 1 plus smoking status, alcohol intake, physical activity, hypertension, BMI and total energy intake.

Statistical tests were two-sided and *P* value of <0.05 was considered statistically significant. No adjustments were made for multiple comparisons.

Supplementary Table 5 Associations between the Kesse-Guyot EAT-Lancet diet index and risks of depression and anxiety after excluding participants who only completed the online 24-hour dietary recall questionnaire on 1 occasion (N=110,130)

	N_{case}/N_{total}	Model 0[1]		Model 1[2]		Model 2[3]	
		HR (95% CI)	*P* value	HR (95% CI)	*P* value	HR (95% CI)	*P* value
Depression							
EAT-Lancet diet index categories							
Quintile 1	517/19626	REF		REF		REF	
Quintile 2	503/23586	0.804 (0.712-0.910)	<0.001	0.779 (0.688-0.881)	<0.001	0.841 (0.743-0.952)	0.006
Quintile 3	484/23936	0.763 (0.674-0.864)	<0.001	0.718 (0.634-0.814)	<0.001	0.802 (0.707-0.910)	<0.001
Quintile 4	523/23179	0.850 (0.753-0.960)	0.009	0.774 (0.683-0.876)	<0.001	0.879 (0.775-0.997)	0.044
Quintile 5	425/19803	0.810 (0.712-0.921)	0.001	0.705 (0.617-0.804)	<0.001	0.806 (0.703-0.924)	0.002
P for trend	-	0.013		<0.001		0.015	
100-point increment in diet score	2452/110130	0.852 (0.753-0.964)	0.011	0.740 (0.651-0.841)	<0.001	0.844 (0.741-0.961)	0.010
Anxiety							
EAT-Lancet diet index categories							
Quintile 1	638/19626	REF		REF		REF	
Quintile 2	690/23586	0.895 (0.804-0.997)	0.044	0.842 (0.755-0.938)	0.002	0.880 (0.790-0.981)	0.021
Quintile 3	712/23936	0.912 (0.819-1.015)	0.090	0.821 (0.737-0.915)	<0.001	0.871 (0.781-0.971)	0.013
Quintile 4	711/23179	0.939 (0.844-1.045)	0.246	0.809 (0.726-0.902)	<0.001	0.860 (0.771-0.961)	0.008
Quintile 5	637/19803	0.986 (0.884-1.101)	0.805	0.806 (0.720-0.902)	<0.001	0.845 (0.752-0.950)	0.005
P for trend	-	0.832		<0.001		0.009	
100-point increment in diet score	3388/110130	1.023 (0.923-1.134)	0.664	0.838 (0.752-0.933)	0.001	0.869 (0.778-0.969)	0.012
Co-occurrence							
EAT-Lancet diet index categories							
Quintile 1	133/19626	REF		REF		REF	
Quintile 2	146/23586	0.909 (0.718-1.149)	0.425	0.870 (0.687-1.101)	0.247	0.948 (0.748-1.202)	0.658
Quintile 3	127/23936	0.780 (0.611-0.994)	0.045	0.720 (0.563-0.921)	0.009	0.805 (0.629-1.032)	0.087
Quintile 4	138/23179	0.872 (0.687-1.107)	0.260	0.774 (0.607-0.987)	0.039	0.869 (0.679-1.112)	0.264
Quintile 5	115/19803	0.853 (0.664-1.094)	0.211	0.718 (0.555-0.928)	0.012	0.794 (0.609-1.036)	0.089
P for trend	-	0.197		0.008		0.069	
100-point increment in diet score	659/110130	0.856 (0.674-1.086)	0.201	0.720 (0.562-0.922)	0.009	0.790 (0.616-1.012)	0.063

[1]Model 0: unadjusted.

[2]Model 1: adjusted for age, sex, Townsend scores and ethnicity.

[3]Model 2: adjusted for model 1 plus smoking status, alcohol intake, physical activity, hypertension, BMI and total energy intake.

Statistical tests were two-sided and *P* value of <0.05 was considered statistically significant. No adjustments were made for multiple comparisons.

Supplementary Table 6 Associations between the Knuppel EAT-Lancet diet index and risks of depression and anxiety using follow-up phases which began at the time of the completion of the latest dietary assessment

	N_{case}/N_{total}	Model 0[1]		Model 1[2]		Model 2[3]	
		HR (95% CI)	*P* value	HR (95% CI)	*P* value	HR (95% CI)	*P* value
Depression							
EAT-Lancet diet index categories							
≤9	812/33707	REF		REF		REF	
=10	1142/54417	0.869 (0.794-0.951)	0.002	0.851 (0.777-0.931)	<0.001	0.900 (0.822-0.986)	0.023
=11	1221/57882	0.872 (0.798-0.953)	0.002	0.827 (0.756-0.905)	<0.001	0.902 (0.824-0.988)	0.027
≥12	648/32863	0.813 (0.733-0.901)	<0.001	0.827 (0.756-0.905)	<0.001	0.818 (0.734-0.911)	<0.001
P for trend	-	<0.001		<0.001		<0.001	
1-point increment in diet score	3823/178869	0.951 (0.925-0.977)	<0.001	0.924 (0.899-0.951)	<0.001	0.954 (0.927-0.982)	0.001
Anxiety							
EAT-Lancet diet index categories							
≤9	973/33707	REF		REF		REF	
=10	1513/54417	0.962 (0.887-1.042)	0.340	0.909 (0.838-0.985)	0.021	0.939 (0.866-1.019)	0.131
=11	1630/57882	0.972 (0.898-1.053)	0.491	0.870 (0.802-0.943)	<0.001	0.912 (0.841-0.989)	0.028
≥12	875/32863	0.919 (0.839-1.007)	0.069	0.771 (0.703-0.846)	<0.001	0.813 (0.740-0.894)	<0.001
P for trend	-	0.125		<0.001		<0.001	
1-point increment in diet score	4991/178869	0.988 (0.965-1.013)	0.343	0.940 (0.917-0.963)	<0.001	0.954 (0.931-0.979)	<0.001
Co-occurrence							
EAT-Lancet diet index categories							
≤9	224/33707	REF		REF		REF	
=10	335/54417	0.925 (0.781-1.096)	0.367	0.887 (0.749-1.052)	0.169	0.937 (0.790-1.111)	0.453
=11	346/57882	0.896 (0.758-1.060)	0.202	0.821 (0.692-0.974)	0.024	0.892 (0.751-1.059)	0.192
≥12	174/32863	0.793 (0.650-0.966)	0.022	0.686 (0.561-0.840)	<0.001	0.753 (0.613-0.925)	0.007
P for trend	-	0.024		<0.001		0.007	
1-point increment in diet score	1079/178869	0.951 (0.903-1.001)	0.055	0.912 (0.865-0.962)	<0.001	0.938 (0.889-0.990)	0.021

[1]Model 0: unadjusted.

[2]Model 1: adjusted for age, sex, Townsend scores and ethnicity.

[3]Model 2: adjusted for model 1 plus smoking status, alcohol intake, physical activity, hypertension, BMI and total energy intake.

Statistical tests were two-sided and *P* value of <0.05 was considered statistically significant. No adjustments were made for multiple comparisons.

Supplementary Table 7 Associations between the Stubbendorff EAT-Lancet diet index and risks of depression and anxiety using follow-up phases which began at the time of the completion of the latest dietary assessment

	N_{case}/N_{total}	Model 0[1]		Model 1[2]		Model 2[3]	
		HR (95% CI)	*P* value	HR (95% CI)	*P* value	HR (95% CI)	*P* value
Depression							
EAT-Lancet diet index categories							
≤17	539/20116	REF		REF		REF	
18-20	960/40218	0.888 (0.799-0.987)	0.028	0.872 (0.784-0.969)	0.011	0.930 (0.836-1.035)	0.183
21-23	1117/55133	0.750 (0.677-0.832)	<0.001	0.724 (0.653-0.803)	<0.001	0.806 (0.726-0.896)	<0.001
24-26	833/42670	0.723 (0.649-0.806)	<0.001	0.681 (0.610-0.760)	<0.001	0.785 (0.702-0.877)	<0.001
≥27	374/20732	0.665 (0.583-0.759)	<0.001	0.614 (0.537-0.702)	<0.001	0.725 (0.633-0.831)	<0.001
P for trend	-	<0.001		<0.001		<0.001	
1-point increment in diet score	3823/178869	0.967 (0.959-0.976)	<0.001	0.961 (0.953-0.970)	<0.001	0.974 (0.966-0.983)	<0.001
Anxiety							
EAT-Lancet diet index categories							
≤17	651/20116	REF		REF		REF	
18-20	1178/40218	0.903 (0.821-0.994)	0.037	0.854 (0.776-0.940)	0.001	0.890 (0.808-0.980)	0.017
21-23	1479/55133	0.825 (0.752-0.904)	<0.001	0.752 (0.685-0.825)	<0.001	0.802 (0.730-0.881)	<0.001
24-26	1144/42670	0.824 (0.748-0.907)	<0.001	0.723 (0.656-0.797)	<0.001	0.784 (0.710-0.866)	<0.001
≥27	539/20732	0.797 (0.711-0.894)	<0.001	0.680 (0.605-0.763)	<0.001	0.744 (0.661-0.836)	<0.001
P for trend	-	<0.001		<0.001		<0.001	
1-point increment in diet score	4991/178869	0.985 (0.978-0.993)	<0.001	0.973 (0.965-0.980)	<0.001	0.980 (0.972-0.988)	<0.001
Co-occurrence							
EAT-Lancet diet index categories							
≤17	148/20116	REF		REF		REF	
18-20	278/40218	0.938 (0.769-1.145)	0.531	0.902 (0.738-1.102)	0.312	0.961 (0.786-1.175)	0.700
21-23	309/55133	0.758 (0.623-0.922)	0.006	0.709 (0.582-0.864)	<0.001	0.787 (0.644-0.961)	0.019
24-26	243/42670	0.770 (0.627-0.944)	0.012	0.696 (0.566-0.857)	<0.001	0.797 (0.646-0.984)	0.035
≥27	101/20732	0.657 (0.510-0.846)	0.001	0.579 (0.448-0.748)	<0.001	0.675 (0.521-0.876)	0.003
P for trend	-	<0.001		<0.001		<0.001	
1-point increment in diet score	1079/178869	0.968 (0.952-0.983)	<0.001	0.958 (0.942-0.974)	<0.001	0.970 (0.954-0.987)	<0.001

[1]Model 0: unadjusted.

[2]Model 1: adjusted for age, sex, Townsend scores and ethnicity.

[3]Model 2: adjusted for model 1 plus smoking status, alcohol intake, physical activity, hypertension, BMI and total energy intake.

Statistical tests were two-sided and *P* value of <0.05 was considered statistically significant. No adjustments were made for multiple comparisons.

Supplementary Table 8 Associations between the Kesse-Guyot EAT-Lancet diet index and risks of depression and anxiety using follow-up phases which began at the time of the completion of the latest dietary assessment

	N_{case}/N_{total}	Model 0[1]		Model 1[2]		Model 2[3]	
		HR (95% CI)	*P* value	HR (95% CI)	*P* value	HR (95% CI)	*P* value
Depression							
EAT-Lancet diet index categories							
Quintile 1	895/35772	REF		REF		REF	
Quintile 2	729/35774	0.810 (0.734-0.893)	<0.001	0.794 (0.720-0.876)	<0.001	0.858 (0.778-0.947)	0.002
Quintile 3	709/35760	0.787 (0.713-0.869)	<0.001	0.755 (0.683-0.834)	<0.001	0.837 (0.758-0.925)	<0.001
Quintile 4	714/35744	0.792 (0.718-0.874)	<0.001	0.738 (0.668-0.815)	<0.001	0.827 (0.748-0.915)	<0.001
Quintile 5	776/35819	0.858 (0.779-0.944)	0.002	0.768 (0.696-0.848)	<0.001	0.850 (0.768-0.942)	0.002
P for trend	-	0.002		<0.001		0.002	
100-point increment in diet score	3823/178869	0.877 (0.804-0.956)	0.003	0.792 (0.725-0.867)	<0.001	0.862 (0.788-0.943)	0.001
Anxiety							
EAT-Lancet diet index categories							
Quintile 1	1092/35772	REF		REF		REF	
Quintile 2	975/35774	0.888 (0.815-0.969)	0.007	0.846 (0.776-0.922)	<0.001	0.889 (0.815-0.969)	0.008
Quintile 3	928/35760	0.845 (0.774-0.922)	<0.001	0.772 (0.707-0.843)	<0.001	0.822 (0.752-0.898)	<0.001
Quintile 4	962/35744	0.876 (0.803-0.956)	0.003	0.766 (0.702-0.837)	<0.001	0.820 (0.750-0.896)	<0.001
Quintile 5	1034/35819	0.941 (0.864-1.024)	0.158	0.777 (0.712-0.848)	<0.001	0.819 (0.749-0.896)	<0.001
P for trend	-	0.153		<0.001		<0.001	
100-point increment in diet score	4991/178869	0.963 (0.893-1.038)	0.328	0.809 (0.748-0.874)	<0.001	0.840 (0.777-0.909)	<0.001
Co-occurrence							
EAT-Lancet diet index categories							
Quintile 1	248/35772	REF		REF		REF	
Quintile 2	220/35774	0.883 (0.736-1.059)	0.179	0.852 (0.710-1.022)	0.085	0.922 (0.768-1.107)	0.382
Quintile 3	189/35760	0.758 (0.627-0.916)	0.004	0.707 (0.584-0.856)	<0.001	0.784 (0.647-0.950)	0.013
Quintile 4	209/35744	0.838 (0.697-1.007)	0.059	0.753 (0.624-0.907)	0.003	0.841 (0.696-1.016)	0.073
Quintile 5	213/35819	0.852 (0.710-1.023)	0.087	0.729 (0.604-0.879)	0.001	0.801 (0.660-0.972)	0.025
P for trend	-	0.068		<0.001		0.015	
100-point increment in diet score	1079/178869	0.869 (0.737-1.024)	0.093	0.753 (0.636-0.892)	0.001	0.814 (0.687-0.964)	0.017

[1]Model 0: unadjusted.

[2]Model 1: adjusted for age, sex, Townsend scores and ethnicity.

[3]Model 2: adjusted for model 1 plus smoking status, alcohol intake, physical activity, hypertension, BMI and total energy intake.

Statistical tests were two-sided and *P* value of <0.05 was considered statistically significant. No adjustments were made for multiple comparisons.

Supplementary Table 9 Associations between the Knuppel EAT-Lancet diet index and risks of depression and anxiety after excluding depression or anxiety cases that occurred within the first five years of follow-up

	N_{case}/N_{total}	Model 0		Model 1		Model 2	
		HR (95% CI)	*P* value	HR (95% CI)	*P* value	HR (95% CI)	*P* value
Depression							
EAT-Lancet diet index categories							
≤9	632/33345	REF		REF		REF	
=10	852/53906	0.832 (0.750-0.922)	<0.001	0.810 (0.731-0.898)	<0.001	0.856 (0.772-0.950)	0.003
=11	954/57431	0.874 (0.790-0.966)	0.009	0.823 (0.743-0.911)	<0.001	0.896 (0.808-0.993)	0.037
≥12	499/32620	0.804 (0.715-0.904)	<0.001	0.722 (0.641-0.814)	<0.001	0.799 (0.707-0.903)	<0.001
P for trend	-	0.003		<0.001		0.003	
1-point increment in diet score	2937/177302	0.953 (0.924-0.984)	0.003	0.925 (0.896-0.955)	<0.001	0.953 (0.923-0.985)	0.004
Anxiety							
EAT-Lancet diet index categories							
≤9	700/33205	REF		REF		REF	
=10	1098/53696	0.968 (0.881-1.065)	0.506	0.904 (0.822-0.994)	0.037	0.936 (0.851-1.030)	0.176
=11	1199/57163	0.993 (0.905-1.090)	0.884	0.872 (0.793-0.958)	0.004	0.917 (0.833-1.008)	0.074
≥12	624/32451	0.909 (0.816-1.013)	0.085	0.749 (0.671-0.836)	<0.001	0.791 (0.708-0.885)	<0.001
P for trend	-	0.188		<0.001		<0.001	
1-point increment in diet score	3621/176515	0.988 (0.960-1.016)	0.394	0.934 (0.907-0.961)	<0.001	0.949 (0.921-0.977)	<0.001
Co-occurrence							
EAT-Lancet diet index categories							
≤9	216/33624	REF		REF		REF	
=10	322/54350	0.921 (0.775-1.094)	0.347	0.879 (0.739-1.045)	0.144	0.928 (0.779-1.104)	0.396
=11	342/57846	0.918 (0.774-1.088)	0.325	0.833 (0.701-0.990)	0.038	0.904 (0.759-1.077)	0.258
≥12	168/32840	0.794 (0.649-0.971)	0.025	0.678 (0.552-0.832)	<0.001	0.742 (0.601-0.915)	0.005
P for trend	-	0.039		<0.001		0.008	
1-point increment in diet score	1048/178660	0.955 (0.906-1.006)	0.085	0.913 (0.865-0.963)	<0.001	0.938 (0.888-0.991)	0.022

[1]Model 0: unadjusted.
[2]Model 1: adjusted for age, sex, Townsend scores and ethnicity.
[3]Model 2: adjusted for model 1 plus smoking status, alcohol intake, physical activity, hypertension, BMI and total energy intake.
Statistical tests were two-sided and *P* value of <0.05 was considered statistically significant. No adjustments were made for multiple comparisons.

Supplementary Table 10 Associations between the Stubbendorff EAT-Lancet diet index and risks of depression and anxiety after excluding depression or anxiety cases that occurred within the first five years of follow-up

	N_{case}/N_{total}	Model 0[1]		Model 1[2]		Model 2[3]	
		HR (95% CI)	*P* value	HR (95% CI)	*P* value	HR (95% CI)	*P* value
Depression							
EAT-Lancet diet index categories							
≤17	388/19841	REF		REF		REF	
18-20	724/39759	0.928 (0.821-1.050)	0.238	0.907 (0.801-1.026)	0.122	0.969 (0.856-1.097)	0.620
21-23	866/54721	0.804 (0.714-0.907)	<0.001	0.771 (0.683-0.870)	<0.001	0.858 (0.760-0.970)	0.014
24-26	667/42369	0.799 (0.705-0.906)	<0.001	0.747 (0.658-0.849)	<0.001	0.860 (0.756-0.978)	0.021
≥27	292/20612	0.717 (0.616-0.835)	<0.001	0.657 (0.563-0.766)	<0.001	0.775 (0.663-0.906)	0.001
P for trend	-	<0.001		<0.001		<0.001	
1-point increment in diet score	2937/177302	0.975 (0.965-0.984)	<0.001	0.968 (0.958-0.978)	<0.001	0.981 (0.971-0.991)	<0.001
Anxiety							
EAT-Lancet diet index categories							
≤17	462/19781	REF		REF		REF	
18-20	843/39608	0.909 (0.812-1.018)	0.100	0.847 (0.756-0.949)	0.004	0.886 (0.791-0.994)	0.039
21-23	1082/54472	0.846 (0.759-0.943)	0.003	0.755 (0.676-0.842)	<0.001	0.808 (0.723-0.903)	<0.001
24-26	839/42154	0.847 (0.756-0.948)	0.004	0.724 (0.645-0.812)	<0.001	0.789 (0.702-0.887)	<0.001
≥27	395/20500	0.818 (0.715-0.936)	0.003	0.677 (0.591-0.776)	<0.001	0.744 (0.648-0.854)	<0.001
P for trend	-	0.001		<0.001		<0.001	
1-point increment in diet score	3621/176515	0.988 (0.979-0.996)	0.006	0.973 (0.964-0.982)	<0.001	0.980 (0.971-0.989)	<0.001
Co-occurrence							
EAT-Lancet diet index categories							
≤17	139/20052	REF		REF		REF	
18-20	269/40107	0.965 (0.786-1.184)	0.733	0.923 (0.751-1.133)	0.443	0.984 (0.801-1.210)	0.882
21-23	296/55125	0.770 (0.630-0.942)	0.011	0.714 (0.583-0.875)	0.001	0.794 (0.646-0.975)	0.028
24-26	247/42642	0.830 (0.674-1.021)	0.078	0.743 (0.602-0.917)	0.006	0.851 (0.687-1.054)	0.140
≥27	97/20734	0.669 (0.516-0.867)	0.002	0.582 (0.448-0.757)	<0.001	0.680 (0.521-0.888)	0.005
P for trend	-	<0.001		<0.001		0.001	
1-point increment in diet score	1048/178660	0.971 (0.956-0.987)	<0.001	0.960 (0.945-0.977)	<0.001	0.973 (0.956-0.989)	0.001

[1]Model 0: unadjusted.

[2]Model 1: adjusted for age, sex, Townsend scores and ethnicity.

[3]Model 2: adjusted for model 1 plus smoking status, alcohol intake, physical activity, hypertension, BMI and total energy intake.

Statistical tests were two-sided and *P* value of <0.05 was considered statistically significant. No adjustments were made for multiple comparisons.

Supplementary Table 11 Associations between the Kesse-Guyot EAT-Lancet diet index and risks of depression and anxiety after excluding depression or anxiety cases that occurred within the first five years of follow-up

	N_{case}/N_{total}	Model 0[1]		Model 1[2]		Model 2[3]	
		HR (95% CI)	*P* value	HR (95% CI)	*P* value	HR (95% CI)	*P* value
Depression							
EAT-Lancet diet index categories							
Quintile 1	662/35293	REF		REF		REF	
Quintile 2	554/35465	0.828 (0.740-0.927)	0.001	0.810 (0.723-0.907)	<0.001	0.875 (0.781-0.980)	0.021
Quintile 3	548/35488	0.818 (0.731-0.916)	<0.001	0.782 (0.698-0.876)	<0.001	0.866 (0.772-0.971)	0.014
Quintile 4	565/35525	0.842 (0.753-0.943)	0.003	0.781 (0.697-0.876)	<0.001	0.875 (0.780-0.982)	0.023
Quintile 5	608/35531	0.909 (0.814-1.015)	0.090	0.811 (0.724-0.908)	<0.001	0.900 (0.801-1.011)	0.076
P for trend	-	0.150		<0.001		0.102	
100-point increment in diet score	2937/177302	0.923 (0.836-1.019)	0.112	0.831 (0.750-0.920)	0.001	0.902 (0.814-0.999)	0.048
Anxiety							
EAT-Lancet diet index categories							
Quintile 1	776/35165	REF		REF		REF	
Quintile 2	707/35316	0.902 (0.815-0.999)	0.047	0.851 (0.768-0.942)	0.002	0.895 (0.808-0.992)	0.034
Quintile 3	686/35332	0.875 (0.789-0.969)	0.011	0.788 (0.710-0.874)	<0.001	0.841 (0.757-0.933)	0.001
Quintile 4	706/35351	0.900 (0.813-0.996)	0.042	0.774 (0.698-0.859)	<0.001	0.831 (0.749-0.923)	<0.001
Quintile 5	746/35351	0.953 (0.862-1.054)	0.350	0.776 (0.700-0.860)	<0.001	0.823 (0.740-0.915)	<0.001
P for trend	-	0.384		<0.001		<0.001	
100-point increment in diet score	3621/ 176515	0.953 (0.872-1.041)	0.284	0.788 (0.718-0.864)	<0.001	0.822 (0.749-0.903)	<0.001
Co-occurrence							
EAT-Lancet diet index categories							
Quintile 1	238/35625	REF		REF		REF	
Quintile 2	207/35729	0.862 (0.715-1.038)	0.118	0.828 (0.687-0.998)	0.047	0.898 (0.744-1.083)	0.260
Quintile 3	195/35756	0.811 (0.671-0.980)	0.031	0.751 (0.621-0.909)	0.003	0.834 (0.688-1.010)	0.064
Quintile 4	205/35781	0.852 (0.707-1.027)	0.093	0.757 (0.626-0.915)	0.004	0.846 (0.698-1.025)	0.087
Quintile 5	203/35769	0.846 (0.702-1.021)	0.081	0.713 (0.589-0.864)	<0.001	0.781 (0.641-0.952)	0.014
P for trend	-	0.098		<0.001		0.014	
100-point increment in diet score	1048/178660	0.853 (0.722-1.008)	0.063	0.728 (0.612-0.865)	<0.001	0.784 (0.660-0.931)	0.006

[1]Model 0: unadjusted.

[2]Model 1: adjusted for age, sex, Townsend scores and ethnicity.

[3]Model 2: adjusted for model 1 plus smoking status, alcohol intake, physical activity, hypertension, BMI and total energy intake.

Statistical tests were two-sided and *P* value of <0.05 was considered statistically significant. No adjustments were made for multiple comparisons.

Supplementary Table 12 Associations of individual food components of the Knuppel EAT-Lancet index with risks of depression and anxiety

	EAT-Lancet index component points		
	0	1	
		HR (95% CI)	*P* value
Depression			
Whole grains	REF	0.962 (0.360-2.570)	0.939
Potatoes	REF	0.895 (0.760-1.053)	0.181
Vegetables	REF	0.871 (0.819-0.926)	<0.001
Fruits	REF	0.860 (0.802-0.922)	<0.001
Dairy	REF	0.842 (0.637-1.115)	0.230
Beef, lamb and pork	REF	1.095 (1.029-1.165)	0.004
Poultry	REF	0.976 (0.898-1.062)	0.576
Eggs	REF	0.982 (0.919-1.049)	0.582
Fish	REF	1.001 (0.842-1.191)	0.990
Dry beans, lentils, peas	REF	0.837 (0.631-1.111)	0.217
Soy foods	REF	0.982 (0.590-1.634)	0.944
Nuts	REF	1.059 (0.922-1.217)	0.419
Unsaturated oils	REF	0.889 (0.743-1.064)	0.199
Added sugar	REF	0.997 (0.921-1.079)	0.938
Anxiety			
Whole grains	REF	0.529 (0.274-1.019)	0.057
Potatoes	REF	0.944 (0.815-1.092)	0.438
Vegetables	REF	0.913 (0.866-0.963)	<0.001
Fruits	REF	0.865 (0.813-0.920)	<0.001
Dairy	REF	0.958 (0.734-1.250)	0.751
Beef, lamb and pork	REF	1.048 (0.993-1.106)	0.087
Poultry	REF	0.960 (0.892-1.033)	0.276
Eggs	REF	1.021 (0.963-1.082)	0.492
Fish	REF	1.028 (0.881-1.200)	0.725
Dry beans, lentils, peas	REF	0.932 (0.714-1.217)	0.606
Soy foods	REF	0.981 (0.631-1.524)	0.932
Nuts	REF	1.013 (0.897-1.144)	0.833
Unsaturated oils	REF	0.901 (0.769-1.056)	0.197
Added sugar	REF	0.941 (0.878-1.009)	0.087
Co-occurrence			
Whole grains	REF	0.338 (0.108-1.056)	0.062
Potatoes	REF	1.152 (0.818-1.623)	0.417
Vegetables	REF	0.853 (0.760-0.958)	0.007
Fruits	REF	0.773 (0.679-0.881)	<0.001
Dairy	REF	0.992 (0.561-1.756)	0.979
Beef, lamb and pork	REF	1.175 (1.045-1.321)	0.007
Poultry	REF	0.955 (0.816-1.117)	0.565
Eggs	REF	0.992 (0.875-1.125)	0.902
Fish	REF	1.391 (0.947-2.043)	0.092
Dry beans, lentils, peas	REF	0.724 (0.434-1.208)	0.217
Soy foods	REF	2.108 (0.525-8.460)	0.293
Nuts	REF	0.992 (0.758-1.299)	0.955
Unsaturated oils	REF	0.906 (0.646-1.271)	0.569
Added sugar	REF	0.955 (0.820-1.111)	0.548

Models were adjusted for age, sex, Townsend scores, ethnicity, smoking status, alcohol intake, physical activity, hypertension, total energy intake, BMI and other individual food components.

Supplementary Table 13 Associations of individual food components of the Stubbendorff EAT-Lancet index with risks of depression and anxiety

	EAT-Lancet index component points							*P* for trend
	0	1		2		3		
		HR (95% CI)	*P* value	HR (95% CI)	*P* value	HR (95% CI)	*P* value	
Depression								
Whole grains	REF	0.992 (0.909-1.083)	0.861	0.946 (0.884-1.013)	0.110	1.166 (0.983-1.382)	0.077	0.333
Potatoes	REF	1.066 (0.340-3.345)	0.912	1.003 (0.323-3.121)	0.995	0.969 (0.312-3.015)	0.957	0.037
Vegetables	REF	0.884 (0.811-0.964)	0.005	0.841 (0.770-0.917)	<0.001	0.815 (0.748-0.888)	<0.001	<0.001
Fruits	REF	0.826 (0.722-0.946)	0.006	0.828 (0.754-0.909)	<0.001	0.860 (0.791-0.935)	<0.001	<0.001
Dairy	REF	0.488 (0.255-0.935)	0.031	0.503 (0.269-0.941)	0.031	0.451 (0.242-0.841)	0.012	<0.001
Beef, lamb and pork	REF	0.956 (0.876-1.043)	0.309	1.038 (0.920-1.171)	0.544	1.084 (1.006-1.168)	0.035	<0.001
Poultry	REF	0.967 (0.769-1.215)	0.773	0.929 (0.737-1.170)	0.530	1.003 (0.804-1.250)	0.982	0.195
Eggs	REF	0.924 (0.823-1.038)	0.181	0.870 (0.760-0.996)	0.043	0.952 (0.877-1.033)	0.236	0.504
Fish	REF	0.801 (0.561-1.143)	0.221	0.806 (0.703-0.924)	0.002	0.873 (0.816-0.934)	<0.001	<0.001
Legumes	REF	0.955 (0.862-1.058)	0.376	1.054 (0.960-1.158)	0.270	1.049 (0.940-1.171)	0.390	0.315
Nuts	REF	0.946 (0.832-1.074)	0.390	1.003 (0.857-1.174)	0.966	1.253 (0.945-1.662)	0.117	0.498
Unsaturated oils	REF	-	-	1.005 (0.839-1.205)	0.953	-	-	-
Added sugar	REF	0.777 (0.684-0.882)	<0.001	0.721 (0.630-0.826)	<0.001	0.721 (0.619-0.839)	<0.001	0.003
Anxiety								
Whole grains	REF	1.048 (0.973-1.129)	0.216	0.992 (0.935-1.052)	0.783	1.132 (0.973-1.318)	0.109	0.832
Potatoes	REF	0.784 (0.322-1.906)	0.591	0.773 (0.321-1.863)	0.566	0.751 (0.312-1.811)	0.524	0.169
Vegetables	REF	0.885 (0.820-0.956)	0.002	0.869 (0.805-0.939)	<0.001	0.869 (0.806-0.937)	<0.001	<0.001
Fruits	REF	0.865 (0.769-0.973)	0.016	0.872 (0.803-0.948)	0.001	0.840 (0.779-0.906)	<0.001	<0.001
Dairy	REF	1.191 (0.488-2.908)	0.702	1.069 (0.443-2.576)	0.882	1.061 (0.441-2.554)	0.895	0.244
Beef, lamb and pork	REF	1.020 (0.947-1.098)	0.604	1.027 (0.926-1.138)	0.617	1.057 (0.990-1.129)	0.097	0.026
Poultry	REF	0.737 (0.612-0.889)	0.001	0.737 (0.611-0.890)	0.002	0.760 (0.636-0.909)	0.003	0.710
Eggs	REF	0.920 (0.830-1.019)	0.109	0.901 (0.803-1.011)	0.077	0.990 (0.921-1.065)	0.788	0.609
Fish	REF	0.874 (0.655-1.168)	0.364	0.887 (0.792-0.993)	0.037	0.931 (0.879-0.987)	0.016	0.002
Legumes	REF	1.016 (0.933-1.106)	0.723	1.052 (0.970-1.141)	0.221	1.021 (0.925-1.126)	0.684	0.342
Nuts	REF	0.990 (0.891-1.100)	0.853	0.993 (0.866-1.137)	0.915	1.130 (0.871-1.464)	0.357	0.657
Unsaturated oils	REF	-	-	0.960 (0.818-1.126)	0.617	-	-	-
Added sugar	REF	0.861 (0.765-0.969)	0.013	0.795 (0.702-0.901)	<0.001	0.762 (0.663-0.875)	<0.001	<0.001
Co-occurrence								
Whole grains	REF	1.065 (0.904-1.254)	0.455	0.977 (0.858-1.112)	0.722	1.664 (1.260-2.199)	<0.001	0.278

Potatoes	REF	0.717 (0.098-5.247)	0.743	0.891 (0.125-6.366)	0.908	0.831 (0.116-5.940)	0.854	0.524
Vegetables	REF	0.915 (0.778-1.077)	0.286	0.852 (0.722-1.006)	0.058	0.807 (0.684-0.951)	0.010	0.007
Fruits	REF	0.680 (0.525-0.882)	0.004	0.760 (0.640-0.903)	0.002	0.696 (0.596 0.813)	<0.001	<0.001
Dairy	REF	0.663 (0.158-2.788)	0.575	0.698 (0.173-2.815)	0.613	0.631 (0.157-2.538)	0.517	0.140
Beef, lamb and pork	REF	1.065 (0.903-1.256)	0.454	1.132 (0.901-1.421)	0.287	1.197 (1.037-1.380)	0.014	0.005
Poultry	REF	0.833 (0.553-1.255)	0.383	0.891 (0.590-1.346)	0.584	0.852 (0.575-1.262)	0.424	0.733
Eggs	REF	0.917 (0.735-1.144)	0.442	0.750 (0.573-0.982)	0.036	0.968 (0.828-1.131)	0.680	0.955
Fish	REF	0.563 (0.251-1.260)	0.162	0.884 (0.689-1.135)	0.333	0.858 (0.754-0.975)	0.019	0.007
Legumes	REF	0.861 (0.704-1.054)	0.147	1.106 (0.929-1.315)	0.257	0.950 (0.766-1.179)	0.642	0.930
Nuts	REF	1.102 (0.879-1.381)	0.401	0.938 (0.689-1.277)	0.685	1.255 (0.736-2.140)	0.404	0.531
Unsaturated oils	REF	-	-	1.004 (0.713-1.414)	0.980	-	-	-
Added sugar	REF	0.965 (0.749-1.244)	0.783	0.845 (0.646-1.107)	0.222	0.832 (0.616-1.122)	0.228	0.072

Models were adjusted for age, sex, Townsend scores, ethnicity, smoking status, alcohol intake, physical activity, hypertension, total energy intake, BMI and other individual food components.

Statistical tests were two-sided and *P* value of <0.05 was considered statistically significant. No adjustments were made for multiple comparisons.

Supplementary Table 14 Associations of individual food components of the Kesse-Guyot EAT-Lancet diet index with risks of depression and anxiety

	1-point increment in the EAT-Lancet index component points	
	HR (95% CI)	*P* value
Depression		
Whole grains	1.138 (0.995-1.302)	0.059
Potatoes	1.046 (0.962-1.138)	0.294
Vegetables	0.972 (0.950-0.993)	0.011
Fruits	0.991 (0.980-1.002)	0.128
Dairy	0.914 (0.828-1.009)	0.075
Beef, lamb and pork	1.010 (0.995-1.026)	0.197
Poultry	1.021 (0.980-1.063)	0.327
Eggs	0.982 (0.965-1.000)	0.050
Fish	1.076 (1.019-1.137)	0.009
Dry beans, lentils, peas	0.946 (0.883-1.014)	0.120
Soy foods	1.058 (0.902-1.241)	0.491
Nuts	0.985 (0.919-1.056)	0.674
Unsaturated oils	1.046 (1.019-1.073)	<0.001
Added sugar	0.936 (0.912-0.960)	<0.001
Anxiety		
Whole grains	0.992 (0.883-1.114)	0.890
Potatoes	1.049 (0.975-1.129)	0.198
Vegetables	0.990 (0.972-1.009)	0.291
Fruits	0.980 (0.971-0.990)	<0.001
Dairy	0.949 (0.869-1.035)	0.235
Beef, lamb and pork	0.998 (0.985-1.012)	0.791
Poultry	0.993 (0.959-1.029)	0.695
Eggs	0.993 (0.977-1.009)	0.393
Fish	1.024 (0.977-1.074)	0.315
Dry beans, lentils, peas	0.963 (0.906-1.024)	0.231
Soy foods	1.119 (0.961-1.302)	0.148
Nuts	0.970 (0.913-1.030)	0.320
Unsaturated oils	1.042 (1.019-1.066)	<0.001
Added sugar	0.943 (0.921-0.964)	<0.001
Co-occurrence		
Whole grains	1.294 (1.003-1.670)	0.047
Potatoes	1.209 (1.027-1.424)	0.023
Vegetables	0.977 (0.938-1.019)	0.283
Fruits	0.975 (0.954-0.996)	0.020
Dairy	0.946 (0.784-1.142)	0.564
Beef, lamb and pork	1.029 (0.999-1.060)	0.057
Poultry	0.991 (0.920-1.068)	0.814
Eggs	0.983 (0.951-1.017)	0.320
Fish	1.094 (0.985-1.215)	0.093
Dry beans, lentils, peas	0.984 (0.860-1.127)	0.820
Soy foods	1.289 (0.863-1.926)	0.214
Nuts	0.955 (0.837-1.091)	0.499
Unsaturated oils	1.051 (1.003-1.103)	0.039
Added sugar	0.939 (0.894-0.985)	0.010

Models were adjusted for age, sex, Townsend scores, ethnicity, smoking status, alcohol intake, physical activity, hypertension, total energy intake, BMI and other individual food components.

Supplementary Table 15 Mediating effects of BMI, CVD, T2D and hypertension in the associations between the EAT-Lancet indexes and risks of depression and anxiety

	Mediation proportion (95% CI), %			
	BMI	CVD	T2D	Hypertension
Knuppel EAT-Lancet index				
Depression	22.10 (14.60, 36.00)	1.88 (0.90, 4.00)	-1.18 (-2.71, 0.00)	-0.50 (-1.71, 0.00)
Anxiety	7.80 (3.29, 16.00)	1.57 (0.76, 3.00)	0.17 (-0.68, 1.00)	0.45 (0.00, 1.00)
Co-occurrence	18.50 (8.61, 60.00)	1.40 (0.49, 5.00)	-0.08 (-1.95, 1.00)	-0.63 (-5.32, 0.00)
Stubbendorff EAT-Lancet index				
Depression	17.80 (13.00, 27.00)	1.24 (0.68, 2.00)	-0.55(-1.17, 0.00)	-0.38 (-0.99, 0.00)
Anxiety	7.50 (3.58, 15.00)	1.32 (0.61, 2.00)	0.01 (-0.47, 1.00)	0.38 (-0.01, 1.00)
Co-occurrence	14.10 (7.89, 31.00)	0.98 (0.38, 3.00)	-0.01 (-0.91, 1.00)	-0.63 (-1.85, 0.00)
Kesse-Guyot EAT-Lancet index				
Depression	23.10 (14.3, 41.00)	1.79 (0.74, 4.00)	-1.50 (-3.71, -1.00)	-0.52 (-1.67, 0.00)
Anxiety	6.12 (2.44, 13.00)	1.20 (0.53, 3.00)	0.18 (-0.62, 1.00)	0.36 (0.00, 1.00)
Co-occurrence	17.40 (7.19, 76.00)	1.31 (0.39, 6.00)	-0.12 (-2.36, 3.00)	-0.06 (-4.57, 0.00)

Models were adjusted for age, sex, Townsend scores, ethnicity, smoking status, alcohol intake, physical activity, total energy intake, and BMI, CVD, T2D and hypertension when these were not considered the potential mediator.

Supplementary Table 16 Subgroup analyses of associations between the Knuppel EAT-Lancet diet index and risks of depression and anxiety

	EAT-Lancet diet index categories							*P* for trend	1-point increment in diet score	*P* for interaction
	≤9	=10		=11		>=12				
		HR (95% CI)	*P* value	HR (95% CI)	*P* value	HR (95% CI)	*P* value			
Depression										
Age										0.116
<60	REF	0.936 (0.845-1.037)	0.207	0.897 (0.808-0.995)	0.041	0.752 (0.662-0.854)	<0.001	<0.001	0.933 (0.903-0.964)	
≥60	REF	0.858 (0.746-0.988)	0.034	0.881 (0.766-1.012)	0.074	0.871 (0.743-1.021)	0.089	0.174	0.973 (0.932-1.016)	
Sex										0.969
Male	REF	0.842 (0.747-0.949)	0.005	0.809 (0.712-0.920)	0.001	0.867 (0.734-1.026)	0.096	0.011	0.945 (0.906-0.986)	
Female	REF	0.981 (0.874-1.102)	0.748	0.970 (0.866-1.086)	0.598	0.815 (0.717-0.926)	0.002	0.002	0.951 (0.920-0.984)	
Townsend deprivation index										0.014
Above median	REF	0.923 (0.829-1.028)	0.146	0.856 (0.766-0.955)	0.006	0.751 (0.659-0.857)	<0.001	<0.001	0.930 (0.899-0.963)	
Others	REF	0.894 (0.785-1.018)	0.091	0.953 (0.838-1.084)	0.462	0.885 (0.761-1.029)	0.112	0.312	0.978 (0.939-1.018)	
Smoking status										0.424
Never	REF	0.943 (0.834-1.067)	0.352	0.944 (0.836-1.067)	0.358	0.821 (0.710-0.948)	0.007	0.015	0.953 (0.918-0.990)	
Previous/current	REF	0.869 (0.776-0.972)	0.014	0.829 (0.739-0.930)	0.001	0.760 (0.663-0.872)	<0.001	<0.001	0.932 (0.899-0.966)	
Anxiety										
Age										0.754
<60	REF	0.949 (0.865-1.041)	0.270	0.912 (0.830-1.001)	0.052	0.813 (0.728-0.908)	<0.001	<0.001	0.952 (0.925-0.980)	
≥60	REF	0.939 (0.831-1.061)	0.312	0.915 (0.811-1.033)	0.150	0.819 (0.713-0.942)	0.005	0.006	0.952 (0.918-0.988)	
Sex										0.546
Male	REF	1.039 (0.929-1.162)	0.503	0.890 (0.788-1.005)	0.060	0.912 (0.778-1.069)	0.256	0.038	0.967 (0.930-1.006)	
Female	REF	0.886 (0.803-0.978)	0.016	0.910 (0.828-1.001)	0.051	0.777 (0.699-0.864)	<0.001	<0.001	0.946 (0.920-0.973)	
Townsend deprivation index										0.001
Above median	REF	0.928 (0.841-1.023)	0.132	0.844 (0.764-0.932)	<0.001	0.731 (0.650-0.822)	<0.001	<0.001	0.923 (0.896-0.952)	
Others	REF	0.976 (0.871-1.092)	0.670	1.011 (0.904-1.130)	0.854	0.942 (0.828-1.072)	0.366	0.590	0.993 (0.959-1.027)	
Smoking status										0.402
Never	REF	0.960 (0.864-1.067)	0.449	0.958 (0.863-1.063)	0.416	0.834 (0.739-0.941)	0.003	0.006	0.961 (0.931-0.992)	
Previous/current	REF	0.924 (0.833-1.026)	0.138	0.857 (0.771-0.953)	0.004	0.787 (0.695-0.891)	<0.001	<0.001	0.938 (0.908-0.970)	
Co-occurrence										
Age										0.036
<60	REF	0.955 (0.786-1.159)	0.641	0.847 (0.693-1.035)	0.105	0.645 (0.501-0.828)	<0.001	<0.001	0.899 (0.844-0.957)	
≥60	REF	0.920 (0.702-1.207)	0.548	0.992 (0.761-1.292)	0.950	0.938 (0.694-1.268)	0.677	0.908	1.002 (0.924-1.086)	
Sex										0.520
Male	REF	0.884 (0.698-1.119)	0.305	0.801 (0.620-1.036)	0.091	1.001 (0.727-1.379)	0.993	0.446	0.966 (0.888-1.050)	
Female	REF	0.987 (0.797-1.223)	0.904	0.958 (0.777-1.181)	0.687	0.697 (0.547-0.887)	0.003	0.003	0.920 (0.865-0.979)	
Townsend deprivation index										0.336
Above median	REF	0.954 (0.776-1.174)	0.657	0.866 (0.700-1.071)	0.183	0.701 (0.543-0.907)	0.007	0.005	0.921 (0.862-0.984)	
Others	REF	0.917 (0.719-1.170)	0.485	0.944 (0.741-1.202)	0.639	0.830 (0.624-1.105)	0.202	0.296	0.959 (0.889-1.035)	
Smoking status										0.667
Never	REF	0.903 (0.715-1.141)	0.394	0.934 (0.742-1.175)	0.558	0.734 (0.557-0.968)	0.029	0.064	0.939 (0.874-1.010)	
Previous/current	REF	0.946 (0.764-1.170)	0.607	0.824 (0.661-1.028)	0.086	0.726 (0.557-0.946)	0.018	0.008	0.916 (0.855-0.981)	

Adjusted for age, sex, Townsend scores, ethnicity, smoking status, alcohol intake, physical activity, hypertension, total energy intake and BMI.
Statistical tests were two-sided and *P* value of <0.05 was considered statistically significant. No adjustments were made for multiple comparisons.

Supplementary Table 17 Subgroup analyses of associations between the Stubbendorff EAT-Lancet diet index and risks of depression and anxiety

	EAT-Lancet diet index categories									*P* for trend	1-point increment in diet score	*P* for interaction
	≤17	18-20		21-23		24-26		>=27				
		HR (95% CI)	*P* value	HR (95% CI)	*P* value	HR (95% CI)	*P* value	HR (95% CI)	*P* value			
Depression												
Age												0.055
<60	REF	0.950 (0.845-1.069)	0.394	0.782 (0.695-0.880)	<0.001	0.751 (0.661-0.852)	<0.001	0.654 (0.557-0.769)	<0.001	<0.001	0.968 (0.959-0.978)	
≥60	REF	0.874 (0.733-1.042)	0.134	0.878 (0.742-1.038)	0.127	0.838 (0.703-0.999)	0.048	0.785 (0.640-0.963)	0.020	0.029	0.983 (0.970-0.996)	
Sex												0.501
Male	REF	0.846 (0.735-0.974)	0.020	0.798 (0.694-0.918)	0.002	0.762 (0.651-0.893)	<0.001	0.781 (0.636-0.960)	0.019	0.001	0.977 (0.964-0.989)	
Female	REF	1.000 (0.871-1.147)	0.998	0.847 (0.741-0.969)	0.016	0.817 (0.711-0.939)	0.005	0.703 (0.597-0.828)	<0.001	<0.001	0.973 (0.963-0.983)	
Townsend deprivation index												<0.001
Above median	REF	0.950 (0.840-1.074)	0.411	0.742 (0.656-0.841)	<0.001	0.727 (0.636-0.830)	<0.001	0.645 (0.545-0.762)	<0.001	<0.001	0.964 (0.953-0.974)	
Others	REF	0.899 (0.765-1.057)	0.198	0.938 (0.804-1.095)	0.419	0.886 (0.753-1.042)	0.143	0.816 (0.673-0.990)	0.039	0.072	0.989 (0.977-1.002)	
Smoking status												0.115
never	REF	0.960 (0.825-1.117)	0.595	0.884 (0.763-1.024)	0.100	0.854 (0.732-0.997)	0.045	0.739 (0.615-0.888)	0.001	<0.001	0.979 (0.967-0.990)	
Previous/current	REF	0.882 (0.775-1.003)	0.056	0.744 (0.655-0.845)	<0.001	0.698 (0.607-0.802)	<0.001	0.659 (0.554-0.784)	<0.001	<0.001	0.966 (0.955-0.976)	
Anxiety												
Age												0.422
<60	REF	0.942 (0.844-1.051)	0.285	0.860 (0.772-0.957)	0.006	0.837 (0.747-0.938)	0.002	0.743 (0.646-0.855)	<0.001	<0.001	0.980 (0.971-0.989)	
≥60	REF	0.826 (0.711-0.959)	0.012	0.789 (0.684-0.911)	0.001	0.746 (0.642-0.866)	<0.001	0.768 (0.648-0.909)	0.002	0.001	0.983 (0.972-0.995)	
Sex												0.937
Male	REF	0.913 (0.798-1.044)	0.184	0.922 (0.808-1.052)	0.228	0.829 (0.715-0.962)	0.014	0.807 (0.665-0.979)	0.030	0.009	0.983 (0.971-0.994)	
Female	REF	0.888 (0.789-0.999)	0.048	0.788 (0.703-0.883)	<0.001	0.782 (0.696-0.879)	<0.001	0.737 (0.644-0.842)	<0.001	<0.001	0.981 (0.972-0.990)	
Townsend deprivation index												<0.001
Above median	REF	0.868 (0.774-0.972)	0.014	0.749 (0.670-0.838)	<0.001	0.703 (0.623-0.793)	<0.001	0.716 (0.621-0.826)	<0.001	<0.001	0.972 (0.963-0.981)	
Others	REF	0.968 (0.840-1.115)	0.652	0.978 (0.854-1.121)	0.754	0.966 (0.839-1.113)	0.637	0.854 (0.723-1.008)	0.062	0.117	0.994 (0.983-1.004)	
Smoking status												0.240
never	REF	0.975 (0.855-1.111)	0.702	0.880 (0.775-0.999)	0.048	0.865 (0.759-0.987)	0.031	0.807 (0.693-0.939)	0.006	<0.001	0.985 (0.975-0.994)	
Previous/current	REF	0.829 (0.734-0.936)	0.003	0.791 (0.703-0.890)	<0.001	0.732 (0.645-0.830)	<0.001	0.710 (0.609-0.829)	<0.001	<0.001	0.975 (0.965-0.985)	
Co-occurrence												
Age												0.022
<60	REF	1.008 (0.806-1.261)	0.944	0.747 (0.594-0.939)	0.012	0.761 (0.597-0.971)	0.028	0.553 (0.399-0.767)	<0.001	<0.001	0.958 (0.939-0.977)	
≥60	REF	0.875 (0.625-1.226)	0.439	0.907 (0.659-1.250)	0.552	0.934 (0.671-1.300)	0.687	0.821 (0.558-1.206)	0.314	0.602	0.992 (0.967-1.017)	
Sex												0.114
Male	REF	0.852 (0.640-1.135)	0.273	0.885 (0.669-1.171)	0.393	1.004 (0.742-1.360)	0.978	0.801 (0.526-1.218)	0.299	0.787	0.990 (0.965-1.015)	
Female	REF	1.021 (0.795-1.310)	0.871	0.755 (0.589-0.967)	0.026	0.746 (0.578-0.964)	0.025	0.603 (0.444-0.819)	0.001	<0.001	0.960 (0.942-0.979)	
Townsend deprivation index												0.014
Above median	REF	0.947 (0.750-1.197)	0.651	0.694 (0.546-0.881)	0.003	0.692 (0.536-0.893)	0.005	0.612 (0.444-0.843)	0.003	<0.001	0.956 (0.937-0.976)	
Others	REF	0.994 (0.729-1.356)	0.971	0.986 (0.731-1.329)	0.926	1.043 (0.766-1.421)	0.788	0.749 (0.511-1.097)	0.138	0.339	0.991 (0.968-1.014)	
Smoking status												0.162
never	REF	1.019 (0.762-1.362)	0.901	0.857 (0.644-1.140)	0.289	0.855 (0.635-1.150)	0.300	0.780 (0.551-1.103)	0.160	0.047	0.979 (0.957-1.001)	
Previous/current	REF	0.885 (0.692-1.130)	0.327	0.731 (0.573-0.933)	0.012	0.760 (0.587-0.984)	0.038	0.491 (0.342-0.705)	<0.001	<0.001	0.956 (0.936-0.977)	

Adjusted for age, sex, Townsend scores, ethnicity, smoking status, alcohol intake, physical activity, hypertension, total energy intake and BMI.

Statistical tests were two-sided and *P* value of <0.05 was considered statistically significant. No adjustments were made for multiple comparisons.

Supplementary Table 18 Subgroup analyses of associations between the Kesse-Guyot EAT-Lancet diet index and risks of depression and anxiety

	EAT-Lancet diet index categories									*P* for trend	1-point increment in diet score	*P* for interaction
	Quintile 1	Quintile 2		Quintile 3		Quintile 4		Quintile 5				
		HR (95% CI)	*P* value	HR (95% CI)	*P* value	HR (95% CI)	*P* value	HR (95% CI)	*P* value			
Depression												
Age												0.002
<60	REF	0.923 (0.826-1.032)	0.158	0.844 (0.752-0.947)	0.004	0.866 (0.771-0.973)	0.015	0.776 (0.687-0.876)	<0.001	<0.001	0.782 (0.705-0.868)	
≥60	REF	0.784 (0.672-0.916)	0.002	0.873 (0.751-1.015)	0.077	0.846 (0.727-0.985)	0.031	0.937 (0.805-1.092)	0.406	0.768	1.013 (0.884-1.160)	
Sex												0.740
Male	REF	0.845 (0.742-0.964)	0.012	0.758 (0.657-0.875)	<0.001	0.863 (0.745-1.000)	0.050	0.874 (0.743-1.029)	0.106	0.028	0.842 (0.729-0.972)	
Female	REF	0.906 (0.799-1.028)	0.125	0.935 (0.827-1.057)	0.282	0.879 (0.778-0.992)	0.037	0.856 (0.758-0.965)	0.011	0.014	0.877 (0.793-0.969)	
Townsend deprivation index												<0.001
Above median	REF	0.827 (0.735-0.931)	0.002	0.829 (0.735-0.934)	0.002	0.772 (0.683-0.872)	<0.001	0.742 (0.656-0.841)	<0.001	<0.001	0.781 (0.701-0.870)	
Others	REF	0.949 (0.825-1.093)	0.471	0.911 (0.790-1.052)	0.203	1.006 (0.873-1.159)	0.932	1.017 (0.878-1.177)	0.824	0.582	1.006 (0.885-1.144)	
Smoking status												0.885
never	REF	0.892 (0.781-1.018)	0.090	0.865 (0.757-0.988)	0.032	0.885 (0.775-1.011)	0.073	0.828 (0.721-0.950)	0.007	0.015	0.850 (0.753-0.958)	
Previous/current	REF	0.838 (0.740-0.948)	0.005	0.826 (0.727-0.937)	0.003	0.800 (0.703-0.910)	<0.001	0.826 (0.726-0.941)	0.004	0.002	0.833 (0.744-0.934)	
Anxiety												
Age												0.393
<60	REF	0.916 (0.829-1.012)	0.084	0.861 (0.778-0.954)	0.004	0.839 (0.756-0.930)	<0.001	0.780 (0.702-0.868)	<0.001	<0.001	0.825 (0.753-0.904)	
≥60	REF	0.906 (0.794-1.033)	0.141	0.879 (0.771-1.002)	0.054	0.902 (0.792-1.027)	0.120	0.887 (0.777-1.013)	0.077	0.127	0.862 (0.768-0.968)	
Sex												0.981
Male	REF	0.941 (0.834-1.062)	0.323	0.908 (0.799-1.033)	0.412	0.864 (0.752-0.992)	0.039	0.900 (0.773-1.049)	0.178	0.044	0.857 (0.750-0.979)	
Female	REF	0.892 (0.802-0.991)	0.034	0.843 (0.760-0.936)	0.001	0.855 (0.773-0.947)	0.003	0.794 (0.718-0.879)	<0.001	<0.001	0.836 (0.768-0.911)	
Townsend deprivation index												0.010
Above median	REF	0.844 (0.758-0.939)	0.002	0.797 (0.715-0.889)	<0.001	0.780 (0.700-0.870)	<0.001	0.739 (0.661-0.825)	<0.001	<0.001	0.778 (0.707-0.856)	
Others	REF	1.010 (0.896-1.139)	0.868	0.969 (0.859-1.094)	0.614	0.986 (0.874-1.114)	0.826	0.958 (0.845-1.086)	0.500	0.440	0.940 (0.843-1.048)	
Smoking status												0.932
never	REF	0.949 (0.848-1.062)	0.361	0.901 (0.805-1.009)	0.071	0.889 (0.794-0.996)	0.042	0.836 (0.744-0.938)	0.002	0.002	0.835 (0.755-0.924)	
Previous/current	REF	0.861 (0.768-0.964)	0.010	0.819 (0.729-0.919)	<0.001	0.818 (0.728-0.919)	<0.001	0.796 (0.707-0.896)	<0.001	0.001	0.827 (0.746-0.917)	
Co-occurrence												
Age												0.006
<60	REF	1.069 (0.871-1.313)	0.523	0.789 (0.629-0.989)	0.040	0.810 (0.646-1.014)	0.066	0.669 (0.527-0.849)	0.001	<0.001	0.678 (0.555-0.829)	
≥60	REF	0.744 (0.547-1.011)	0.059	0.946 (0.710-1.260)	0.702	1.052 (0.794-1.394)	0.722	1.074 (0.806-1.431)	0.625	0.142	1.154 (0.900-1.479)	
Sex												0.752
Male	REF	0.810 (0.623-1.053)	0.115	0.710 (0.531-0.949)	0.021	1.029 (0.781-1.355)	0.840	0.918 (0.666-1.264)	0.600	0.785	0.901 (0.679-1.196)	
Female	REF	1.077 (0.856-1.355)	0.526	0.949 (0.753-1.196)	0.658	0.877 (0.697-1.104)	0.265	0.812 (0.646-1.022)	0.076	0.012	0.800 (0.663-0.965)	
Townsend deprivation index												0.051
Above median	REF	0.850 (0.678-1.065)	0.158	0.739 (0.583-0.936)	0.012	0.823 (0.654-1.036)	0.097	0.710 (0.559-0.902)	0005	0.008	0.825 (0.673-1.012)	
Others	REF	1.100 (0.845-1.432)	0.477	1.014 (0.774-1.328)	0.922	1.031 (0.786-1.352)	0.827	1.009 (0.761-1.336)	0.952	0.865	0.847 (0.663-1.082)	
Smoking status												0.707
never	REF	0.880 (0.684-1.131)	0.318	0.849 (0.660-1.093)	0.204	0.857 (0.666-1.104)	0.233	0.779 (0.599-1.012)	0.061	0.082	0.846 (0.673-1.064)	
Previous/current	REF	0.970 (0.769-1.224)	0.798	0.796 (0.621-1.020)	0.071	0.874 (0.686-1.113)	0.273	0.806 (0.627-1.036)	0.092	0.059	0.776 (0.625-0.963)	

Adjusted for age, sex, Townsend scores, ethnicity, smoking status, alcohol intake, physical activity, hypertension, total energy intake and BMI.

Statistical tests were two-sided and *P* value of <0.05 was considered statistically significant. No adjustments were made for multiple comparisons.

Supplementary Table 19 Baseline characteristics of the analysis population and the total population

Characteristics	Participants selected	All	*P* value
N	180446	428787	
Age (years)	56.16±7.97	56.56±8.13	<0.001
Sex (male, %)	83824 (46.45)	203942 (47.56)	<0.001
Ethnic (%)			<0.001
White	172018 (95.33)	402233 (93.81)	
Mixed	1057 (0.59)	2473 (0.58)	
Asian or British	2573 (1.43)	8860 (2.07)	
Black or Black British	2278 (1.26)	7380 (1.72)	
Other	1862 (1.03)	5467 (1.27)	
Townsend score (%)	-1.63±2.84	-1.37±3.05	<0.001
Smoking status (%)			<0.001
Never smoked	103269 (57.23)	237445 (55.38)	
Previous smoker	63446 (35.16)	146954 (34.27)	
Current smoker	13267 (7.35)	41903 (9.77)	
Alcohol intake (%)			<0.001
Never	10376 (5.75)	31547 (7.36)	
Special occasions only	16955 (9.40)	46765 (10.91)	
One to three times a month	19500 (10.81)	46830 (10.92)	
Once or twice a week	45303 (25.11)	112087 (26.14)	
Three or four times a week	46353 (25.69)	101908 (23.77)	
Daily or almost daily	41828 (23.18)	88423 (20.62)	
Physical activity (%)			<0.001
None	2216 (1.23)	6337 (1.48)	
Low	24885 (13.79)	55615 (12.97)	
Moderate	82185 (45.55)	175802 (41.00)	
High	44204 (24.50)	107557 (25.08)	
Hypertension (%)	76077 (42.16)	190911 (44.52)	<0.001
Body mass index (kg/m2)	26.82±4.53	27.30±4.68	<0.001

Data were presented as frequency (%) or mean ± standard deviation.

Statistical tests were two-sided and *P* value of <0.05 was considered statistically significant. No adjustments were made for multiple comparisons.

Supplementary Table 20 Associations between the EAT-Lancet indexes and risks of depression and anxiety in White

	Depression		Anxiety		Co-occurrence	
	HR (95% CI)	*P* value	HR (95% CI)	*P* value	HR (95% CI)	*P* value
Knuppel EAT-Lancet index						
≤9	REF		REF		REF	
=10	0.904 (0.830-0.984)	0.019	0.953 (0.883-1.028)	0.209	0.928 (0.790-1.090)	0.360
=11	0.891 (0.818-0.970)	0.008	0.917 (0.850-0.989)	0.025	0.880 (0.748-1.036)	0.125
≥12	0.792 (0.715-0.877)	<0.001	0.823 (0.753-0.899)	<0.001	0.753 (0.619-0.915)	0.004
P for trend	<0.001		<0.001		0.004	
1-point increment in diet score	0.945 (0.920-0.971)	<0.001	0.954 (0.932-0.977)	<0.001	0.935 (0.888-0.984)	0.010
Stubbendorff EAT-Lancet index						
≤17	REF		REF		REF	
18-20	0.927 (0.839-1.025)	0.141	0.914 (0.834-1.001)	0.054	0.964 (0.795-1.169)	0.709
21-23	0.817 (0.740-0.901)	<0.001	0.848 (0.776-0.926)	<0.001	0.813 (0.672-0.984)	0.033
24-26	0.783 (0.705-0.870)	<0.001	0.822 (0.748-0.902)	<0.001	0.832 (0.681-1.016)	0.071
≥27	0.707 (0.622-0.804)	<0.001	0.779 (0.697-0.870)	<0.001	0.668 (0.521-0.858)	0.002
P for trend	<0.001		<0.001		<0.001	
1-point increment in diet score	0.973 (0.965-0.981)	<0.001	0.983 (0.976-0.990)	<0.001	0.972 (0.957-0.987)	<0.001
Kesse-Guyot EAT-Lancet index						
Quintile 1	REF		REF		REF	
Quintile 2	0.855 (0.779-0.937)	<0.001	0.910 (0.839-0.987)	0.023	0.918 (0.771-1.093)	0.335
Quintile 3	0.850 (0.774-0.933)	<0.001	0.882 (0.813-0.958)	0.003	0.836 (0.698-1.001)	0.051
Quintile 4	0.847 (0.771-0.931)	<0.001	0.868 (0.799-0.943)	<0.001	0.890 (0.745-1.064)	0.202
Quintile 5	0.826 (0.750-0.910)	<0.001	0.826 (0.759-0.900)	<0.001	0.812 (0.674-0.978)	0.028
P for trend	<0.001		<0.001		0.035	
100-point increment in diet score	0.849 (0.779-0.925)	<0.001	0.850 (0.789-0.916)	<0.001	0.830 (0.706-0.977)	0.025

Adjusted for age, sex, Townsend scores, ethnicity, smoking status, alcohol intake, physical activity, hypertension, total energy intake and BMI.

Statistical tests were two-sided and *P* value of <0.05 was considered statistically significant. No adjustments were made for multiple comparisons.

Supplementary Table 21 Definition of portion size and food items used in this study

Component	Definition of 1 portion	Food items based on the Oxford WebQ questionnaire
**Whole grains**		
1 Rice, wheat, corn, and other	1 portion: 130 g	porridge, muesli, oat crunch, sweetened cereal, plain cereal, bran cereal, whole-wheat cereal, other cereal, white pasta, wholemeal pasta, white rice, brown rice, sushi, snackpot, couscous, other grain
**Tubers and starchy vegetables**		
2 Potatoes	1 portion: 58 g	fried, boiled, baked or mashed potatoes, sweet potato
**Vegetables**		
3 All vegetables	1 portion: 100 g	quorn, mixed vegetable, vegetable pieces, coleslaw, side salad, avocado, broad bean, green bean, beetroot, broccoli, butternut squash, cabbage, carrot, cauliflower, celery, courgette, cucumber, garlic, leek, lettuce, mushroom, onion, parsnip, pea, sweet pepper, spinach, fresh tomato, tinned tomato, turnip, watercress, other vegetable, sweetcorn
**Fruits**		
4 All fruits	1 portion: 150 g	stewed fruit, prune (1 portion: 48g), dried fruit (1 portion: 48g), mixed fruit, apple, banana, berry (1 portion: 48g), cherry (1 portion: 48g), grapefruit, grape (1 portion: 48g), mango, melon, orange, satsuma, peach, pear, pineapple, plum (1 portion: 48g), other fruit
**Dairy foods**		
5 Whole milk or derivative equivalents	1 portion: 258 g	milk, favoured milk, yogurt (1 portion: 180g), low fat hard cheese, hard cheese, soft cheese, blue cheese, low fat cheese spread, cheese spread, cottage cheese, feta, mozzarella, goat's cheese, other cheese (1 portion: 28g)
**Protein sources**		
6 Beef, lamb, pork		bacon, ham (1 portion: 40g), liver, sausage, beef, pork, lamb (1 portion: 80g)
7 Chicken, other poultry	1 portion: 80 g	crumbed or deep-fried poultry, poultry
8 Eggs	1 portion: 60 g	whole egg, omelette, eggs in sandwich, scotch egg, other egg
9 Fish	1 portion: 100 g	shellfish, tinned tuna, oily fish, breaded fish, battered fish, white fish, other fish
Legumes		
10 Dry beans, lentils, peas	1 portion: 100 g	dried lentils, peas and baked beans
11 Soy foods	1 portion: 100 g	tofu
12 Peanuts or tree nuts	1 portion: 28 g	unsalted peanuts, salted nuts, unsalted nuts, seed
**Added fats**		
13 Palm oil, unsaturated oils, dairy fats (incl. in milk), lard or tallow		ratio of 0.8 for unsaturated: saturated fat intake*
**Added sugars**		
14 All sweeteners		derived from sweet foods and beverages, and sugar added to food or beverages recorded in the 24-hour diet recall

*Too little information about the types of oils were recorded in the 24-hour diet recall so fat intake was approximated as a ratio of total unsaturated and saturated fat in line with the EAT-Lancet reference diet[Reference 4].

Supplementary Table 22 Cut-offs for each component of the Knuppel and the Kesse-Guyot EAT-Lancet diet index

Component	Criteria for minimum score of 0	Criteria for maximum score of 1*
**Whole grains**		
1 Rice, wheat, corn, and other	>464 g/day	≤464 g/day
**Tubers and starchy vegetables**		
2 Potatoes	>100 g/day	≤100 g/day
**Vegetables**		
3 All vegetables	<200 g/day	≥200 g/day
**Fruits**		
4 All fruits	<100 g/day	≥100 g/day
**Dairy foods**		
5 Whole milk or derivative equivalents	>500g/day	≤500 g/day
**Protein sources**		
6 Beef, lamb, pork	>28 g/day	≤28 g/day
7 Chicken, other poultry	>58 g/day	≤58 g/day
8 Eggs	>25 g/day	≤25 g/day
9 Fish	>100g/day	≤100g/day
Legumes		
10 Dry beans, lentils, peas	>100 g/day	≤100g/day
11 Soy foods	>50 g/day	≤50g/day
12 Peanuts or tree nuts	<25 g/day	≥25g/day
**Added fats**		
13 Palm oil, unsaturated oils, dairy fats (incl. in milk), lard or tallow		Ratio of 0.8 for unsaturated: saturated fat intake
**Added sugars**		
14 All sweeteners	>31g/day	≤31g/day

*Each food component contributed 1 point if participants met the recommendation of the EAT-Lancet diet pattern.

Supplementary Table 23 Cut-offs for each component of the Stubbendorff EAT-Lancet diet index

Food components in the EAT-Lancet diet index		Target intake (reference interval)	3 points	2 points	1 point	0 point
Emphasized intake	Vegetables	300 (200–600)	>300	200–300	100–200	<100
	Fruits	200 (100–300)	>200	100–200	50–100	<50
	Unsaturated oils	40 (20–80)	>40	20–40	10–20	<10
	Legumes	75 (0–150)	>75	37.5–75	18.75–37.5	<18.75
	Nuts	50 (0–100)	>50	25–50	12.5–25	<12.5
	Whole grains*	232	>232	116–232	58–116	<58
	Fish	28 (0–100)	>28	14–28	7–14	<7
Limited intake	Beef and lamb	7 (0–14)	<7	7–14	14–28	>28
	Pork	7 (0–14)	<7	7–14	14–28	>28
	Poultry	29 (0–58)	<29	29–58	58–116	>116
	Eggs	13 (0–25)	<13	13–25	25–50	>50
	Dairy**	250 (0–500)	<250	250–500	500–1000	>1000
	Potatoes	50 (0–100)	<50	50–100	100–200	>200
	Added sugar	31 (0–31)	<31	31–62	62–124	>124

* Definition of whole grains in the Stubbendorff EAT-Lancet diet index excludes refined products.

** Intakes of different dairy products were weighted differently according to [Reference 7].

Supplementary Table 24 Proportion of 180,446 participants adhering to the Knuppel EAT-Lancet diet index recommendations

Component	Recommendation met n (%)
**Whole grains**	
1 Rice, wheat, corn, and other	180293 (99.92)
**Tubers and starchy vegetables**	
2 Potatoes	175016 (96.99)
**Vegetables**	
3 All vegetables	99974 (55.40)
**Fruits**	
4 All fruits	142224 (78.82)
**Dairy foods**	
5 Whole milk or derivative equivalents	178952 (99.17)
**Protein sources**	
6 Beef, lamb, pork	76571 (42.43)
7 Chicken, other poultry	154742 (85.76)
8 Eggs	132242 (73.29)
9 Fish	175171 (97.08)
Legumes	
10 Dry beans, lentils, peas	178887 (99.14)
11 Soy foods	179885 (99.69)
12 Peanuts or tree nuts	8288 (4.59)
**Added fats**	
13 Palm oil, unsaturated oils, dairy fats (incl. in milk), lard or tallow	176293 (97.70)
**Added sugars**	
14 All sweeteners	34272 (18.99)

Supplementary Table 25 Proportion of 180,446 participants adhering to the Stubbendorff EAT-Lancet diet index recommendations

Component	0 n(%)	1 n(%)	2 n(%)	3 n(%)
Whole grains	85396 (47.32)	28190 (15.62)	61762 (34.23)	5098 (2.83)
Potatoes	111 (0.06)	5319 (2.95)	48143 (26.68)	126873 (70.31)
Vegetables	38752 (21.48)	41720 (23.12)	44672 (24.76)	55302 (30.65)
Fruits	26368 (14.61)	11854 (6.57)	40569 (22.48)	101655 (56.34)
Dairy	148 (0.08)	3427 (1.90)	30002 (16.63)	146869 (81.39)
Beef, lamb and pork*	67336 (37.72)	36621 (20.29)	14915 (8.27)	61574 (34.12)
Poultry	2856 (1.58)	22848 (12.66)	27178 (15.06)	127564 (70.69)
Eggs	26771 (14.84)	21433 (11.88)	15534 (8.61)	116708 (64.71)
Fish	102199 (56.64)	1649 (0.91)	12068 (6.69)	64530 (35.76)
Legumes	126742 (70.24)	19965 (11.06)	20516 (11.37)	13223 (7.33)
Nuts	160468 (88.93)	11690 (6.48)	6766 (3.75)	1522 (0.84)
Unsaturated oils**	4153 (2.30)	NA	176293 (97.70)	NA
Added sugar	8777 (4.86)	64198 (35.58)	73199 (40.57)	34272 (18.99)

* The ability of 24-h dietary recall questionnaire to separate red meat into beef/lamb and pork was limited hence these were aggregated into total red meat intake.

** Too little information about the types of oils were recorded in the 24-hour diet recall so fat intake was approximated as a ratio of total unsaturated and saturated fat. Participants who met the recommendation of ratio of 0.8 for unsaturated:saturated fat intake were assigned with 2 points and those did not meet the recommendation were assigned with 0 point.

Supplementary Table 26 Scores of each individual components of the Kesse-Guyot EAT-Lancet diet index

Component	Score
**Whole grains**	
1 Rice, wheat, corn, and other	67.29 (53.45-82.16)
**Tubers and starchy vegetables**	
2 Potatoes	65.87 (39.05-100.00)
**Vegetables**	
3 All vegetables	30.76 (-37.02-120.92)
**Fruits**	
4 All fruits	190.87 (50.90-388.65)
**Dairy foods**	
5 Whole milk or derivative equivalents	88.19 (63.21-98.10)
**Protein sources**	
6 Beef, lamb, pork	-74.38 (-225.70-100.00)
7 Chicken, other poultry	100.00 (21.56-100.00)
8 Eggs	100.00 (-30.54-100.00)
9 Fish	100.00 (40.64-100.00)
Legumes	
10 Dry beans, lentils, peas	100.00 (69.01-100.00)
11 Soy foods	100.00 (100.00-100.00)
12 Peanuts or tree nuts	-100.00 (-100.00- -100.00)
**Added fats**	
13 Palm oil, unsaturated oils, dairy fats (incl. in milk), lard or tallow	136.02 (80.00-216.37)
**Added sugars**	
14 All sweeteners	-119.63 (-192.59- -56.13)

Scores were presented as median (interquartile range).

References

[1] Knuppel A, Papier K, Key TJ, Travis RC. EAT-Lancet score and major health outcomes: the EPIC-Oxford study. Lancet **394**, 213-214 (2019).

[2] Ministry of Agriculture Fisheries and Food. Food Portion Sizes. 2nd ed. London: HMSO (1993).

[3] Public Health England. Composition of Foods Integrated Dataset (CoFID). (2019). Available online at: https://www.gov.uk/government/publications/composition-of-foods-integrated-dataset-cofid (accessed January 22, 2021).

[4] Xu C, Cao Z, Yang H, Hou Y, Wang X, Wang Y. Association Between the EAT-Lancet Diet Pattern and Risk of Type 2 Diabetes: A Prospective Cohort Study. Front Nutr **8**, 784018 (2022).

[5] Willett W, Rockström J, Loken B, et al. Food in the Anthropocene: the EAT-Lancet Commission on healthy diets from sustainable food systems. Lancet **393**, 447-492 (2019).

[6] Zhang S, Stubbendorff A, Olsson K, et al. Adherence to the EAT-Lancet diet, genetic susceptibility, and risk of type 2 diabetes in Swedish adults. Metabolism **141**, 155401 (2023).

[7] Stubbendorff A, Sonestedt E, Ramne S, Drake I, Hallström E, Ericson U. Development of an EAT-Lancet index and its relation to mortality in a Swedish population. Am J Clin Nutr **115**, 705-716 (2022).

[8] Emmanuelle Kesse-Guyot, Pauline Rebouillat, Joséphine Brunin, et al. Environmental and nutritional analysis of the EAT-Lancet diet at the individual level: insights from the NutriNet-Santé study. Journal of Cleaner Production **296**, 126555 (2021).

[9] Wu H, Gu Y, Meng G, et al. Quality of plant-based diet and the risk of dementia and depression among middle-aged and older population. Age Ageing **52**, afad070 (2023).

[10] Fung TT, Chiuve SE, McCullough ML, Rexrode KM, Logroscino G, Hu FB. Adherence to a DASH-style diet and risk of coronary heart disease and stroke in women. Arch Intern Med **168**, 713-720 (2008).

[11] Martínez-González MÁ, Corella D, Salas-Salvadó J, et al. Cohort profile: design and methods of the PREDIMED study. Int J Epidemiol **41**, 377-385 (2012).

[12] Shannon OM, Ranson JM, Gregory S, et al. Mediterranean diet adherence is associated with lower dementia risk, independent of genetic predisposition: findings from the UK Biobank prospective cohort study. BMC Med **21**, 81 (2023).

[13] Jarman B, Townsend P, Carstairs V. Deprivation indices[J]. BMJ: British Medical Journal **303**, 523 (1991).

[14] Ainsworth B E, Haskell W L, Whitt M C, et al. Compendium of physical activities: an update of activity codes and MET intensities[J]. Medicine and science in sports and exercise **32**, S498-S504 (2000).

[15] The IPAQ Group. IPAQ scoring protocol - International Physical Activity Questionnaire.
