# Supplementary material for: Adherence to the EAT-Lancet diet and incident depression and anxiety
Source: Nat Commun. 2024 Jul 3;15:5599. doi: 10.1038/s41467-024-49653-8 (PMC11222463; doi:10.1038/s41467-024-49653-8)
Supplement: Supplementary file 2 — Reporting Summary [file 41467_2024_49653_MOESM2_ESM.pdf]

nature portfolio

Corresponding author(s): Chaofu Ke

Last updated by author(s): Apr 29, 2024

Reporting Summary

Nature Portfolio wishes to improve the reproducibility of the work that we publish. This form provides structure for consistency and transparency in reporting. For further information on Nature Portfolio policies, see our Editorial Policies and the Editorial Policy Checklist.

Statistics

For all statistical analyses, confirm that the following items are present in the figure legend, table legend, main text, or Methods section.

n/a	Confirmed	
☐	☒	The exact sample size (*n*) for each experimental group/condition, given as a discrete number and unit of measurement
☐	☒	A statement on whether measurements were taken from distinct samples or whether the same sample was measured repeatedly
☐	☒	The statistical test(s) used AND whether they are one- or two-sided<br>*Only common tests should be described solely by name; describe more complex techniques in the Methods section.*
☐	☒	A description of all covariates tested
☐	☒	A description of any assumptions or corrections, such as tests of normality and adjustment for multiple comparisons
☐	☒	A full description of the statistical parameters including central tendency (e.g. means) or other basic estimates (e.g. regression coefficient) AND variation (e.g. standard deviation) or associated estimates of uncertainty (e.g. confidence intervals)
☐	☒	For null hypothesis testing, the test statistic (e.g. *F*, *t*, *r*) with confidence intervals, effect sizes, degrees of freedom and *P* value noted<br>*Give P values as exact values whenever suitable.*
☒	☐	For Bayesian analysis, information on the choice of priors and Markov chain Monte Carlo settings
☒	☐	For hierarchical and complex designs, identification of the appropriate level for tests and full reporting of outcomes
☐	☒	Estimates of effect sizes (e.g. Cohen's *d*, Pearson's *r*), indicating how they were calculated

Our web collection on statistics for biologists contains articles on many of the points above.

Software and code

Policy information about availability of computer code

Data collection	The data collection of this study used questionnaires and physical measurements. The collected information was imported into SAS ver 9.4 to save as SAS datasets.
Data analysis	Statistical analysis were performed using the SAS version 9.4 (SAS Institute) and R (version 4.1.1 software). The codes are available at http://github.com/Luxujia/UKB_EAT-Lancet_and_Depression_and_Anxiety.

For manuscripts utilizing custom algorithms or software that are central to the research but not yet described in published literature, software must be made available to editors and reviewers. We strongly encourage code deposition in a community repository (e.g. GitHub). See the Nature Portfolio guidelines for submitting code & software for further information.

Data

Policy information about availability of data

All manuscripts must include a data availability statement. This statement should provide the following information, where applicable:

- Accession codes, unique identifiers, or web links for publicly available datasets
- A description of any restrictions on data availability
- For clinical datasets or third party data, please ensure that the statement adheres to our policy

Data are available in a public, open access repository. This research has been conducted using the UK Biobank Resource under Application Number 60651. The data that supported the findings of this study are available on application to the UK Biobank team at http://WWW.ukbiobank.ac.uk/. Source data are provided with this paper.

Research involving human participants, their data, or biological material

Policy information about studies with human participants or human data. See also policy information about sex, gender (identity/presentation), and sexual orientation and race, ethnicity and racism.

Reporting on sex and gender	The reported sex of participants was accessed through the UK Biobank data-field 31. Sex was included as a covariate in the statistical analysis. Sex proportion was reported and one of the subgroup analysis was stratified by sex. Gender was not considered in this study.
Reporting on race, ethnicity, or other socially relevant groupings	The reported ethnicity and Townsend deprivation index of participants were accessed through the UK Biobank data-field 21000 and 22189, respectively. Ethnicity and Townsend deprivation index were included as a covariate in the statistical analyses. Ethnicity proportion and the mean (SD) of the Townsend deprivation index were reported. One of the subgroup analyses was performed stratified by Townsend deprivation index and the main analysis was repeated in White.
Population characteristics	The mean age (SD) of participants in this study was 56.2 (8.0) years at baseline and 46.45% were male. Over 95% of participants were White. During a median (IQR) of follow-up of 11.62 (11.00-12.43) years, 4548, 6026, 1262 incident cases of depression, anxiety and the co-occurrence of depression and anxiety were identified.
Recruitment	The UK Biobank is a large and prospective study with over 500,000 participants aged 37-73 years (99.5% aged 40-69 years) recruited from the general population between 2006-2009. After providing written infomed consent, participants completed a touch screen questionnaire and a face-to-face interview and underwent a series of physical measurements and biological sample collection. The 24-hour dietary questionnaire was first introduced as a part of assessment visit towards the end of recruitment for the last 70,000 participants (2009-2010), and participants were invited to complete 4 addtional questionnaires online at 3-4 monthly intervals on four separate occassions between Feb 2011 and April 2012.
Ethics oversight	UK Biobank research has recieved approval from the North West Multicenter Research Ethical Committee. Written informed consents were provided by all participants.

Note that full information on the approval of the study protocol must also be provided in the manuscript.

Field-specific reporting

Please select the one below that is the best fit for your research. If you are not sure, read the appropriate sections before making your selection.

☒ Life sciences ☐ Behavioural & social sciences ☐ Ecological, evolutionary & environmental sciences

For a reference copy of the document with all sections, see nature.com/documents/nr-reporting-summary-flat.pdf

Life sciences study design

All studies must disclose on these points even when the disclosure is negative.

Sample size	We chose this sample size because of the data availability. In this study, we included 180,446 participants with available data on measures of adherence to the EAT-Lancet diet and potential covariates. According to the rule-of-thumb estimation, at least ten events are required per variable (including dummy variable) in the model (Riley et al. BMJ. 2020). In the fully adjusted model, we included a total of 42 variables (includiing dummy variables) in the Cox regression models. Thus, at least 420 events for each outcome were required. The events for incident depression, anxiety and their co-occurrence were 4548, 6026 and 1262, respectively. Therefore, the sample size of this study should be sufficient.
Data exclusions	In this study, we included participants with available data on measures of adherence to the EAT-Lancet diet and potential covariates. We excluded participants who met any of the following criteria: (1) withdrew from the survey; (2) suffered from depression or anxiety at baseline; (3) reported use of anxiolytics or antidepressant at baseline; (4) with abnormal total energy intakes.
Replication	After data exclusions, individuals who completed at least 1 dietary assessment were included in the main analysis (N= 180,446). We repeated the analysis among participants with 2 or more dietary assessments (N= 110,130) and the replication was successful. This is a population-based epidemiology cohort study, the current findings may need to be further validated by clinical traits and/or cohorts of different settings and populations.

Randomization	This is an observational study where researched observes the events and do not control them. It does not require randomization to allocate patients and controls. The covariates were controlled by using the regression models which included all potential covariates.
Blinding	This is a population-based observational cohort study which does not perform binding to give treatments or placebo as randomized controlled trials.

Reporting for specific materials, systems and methods

We require information from authors about some types of materials, experimental systems and methods used in many studies. Here, indicate whether each material, system or method listed is relevant to your study. If you are not sure if a list item applies to your research, read the appropriate section before selecting a response.

Materials & experimental systems

n/a	Involved in the study
☒	☐ Antibodies
☒	☐ Eukaryotic cell lines
☒	☐ Palaeontology and archaeology
☒	☐ Animals and other organisms
☒	☐ Clinical data
☒	☐ Dual use research of concern
☒	☐ Plants

Methods

n/a	Involved in the study
☒	☐ ChIP-seq
☒	☐ Flow cytometry
☒	☐ MRI-based neuroimaging

Plants

Seed stocks	*Report on the source of all seed stocks or other plant material used. If applicable, state the seed stock centre and catalogue number. If plant specimens were collected from the field, describe the collection location, date and sampling procedures.*
Novel plant genotypes	*Describe the methods by which all novel plant genotypes were produced. This includes those generated by transgenic approaches, gene editing, chemical/radiation-based mutagenesis and hybridization. For transgenic lines, describe the transformation method, the number of independent lines analyzed and the generation upon which experiments were performed. For gene-edited lines, describe the editor used, the endogenous sequence targeted for editing, the targeting guide RNA sequence (if applicable) and how the editor was applied.*
Authentication	*Describe any authentication procedures for each seed stock used or novel genotype generated. Describe any experiments used to assess the effect of a mutation and, where applicable, how potential secondary effects (e.g. second site T-DNA insertions, mosiacism, off-target gene editing) were examined.*
